# Mirror-Image RNA: A Right-Handed Z-Form RNA and Its Ligand Complex

**DOI:** 10.3390/molecules29204900

**Published:** 2024-10-16

**Authors:** Yi Song, Shiyu Wang, Yan Xu

**Affiliations:** Division of Chemistry, Department of Medical Sciences, Faculty of Medicine, University of Miyazaki, 5200 Kihara, Kiyotake, Miyazaki 889-1692, Japan

**Keywords:** mirror image, right hand, Z-form L-RNA, RNA-ligand complex

## Abstract

Until now, Z-form RNAs were believed to only adopt a left-handed double-helix structure. In this study, we describe the first observation of a right-handed Z-form RNA in NMR solution formed by L-nucleic acid RNA and present the first resolution of structure of the complex between a right-handed Z-form RNA and a curaxin ligand. These results provide a platform for the design of topology specific to Z-form-targeting compounds and are valuable for the development of new potent anticancer drugs.

## 1. Introduction

**Scheme 1:**
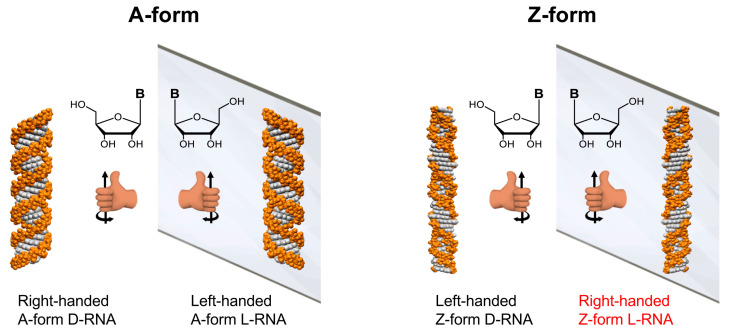
Illustration of mirror-image RNA. The left column displays a right-handed helix A-form D-RNA and its mirror-image, a left-handed A-form L-RNA consisting of L-nucleic acids. The right column shows the left-handed helix Z-form D-RNA and its mirror-image with a right-handed Z-form L-RNA consisting of L-nucleic acids (The words in red highlight the typical structure, which is the main focus of this study).

Nucleic acids have exquisitely evolved to store genetic information, primarily composed of D-nucleic acids, folding into the most common secondary structure with right-handed double-helical conformational features, fundamentally recognized as A-RNA or B-DNA (Scheme 1, left column). The right-handed A-form RNA plays a crucial role in cellular processes and functions, such as responding to viral infections, post-transcriptional gene silencing, RNA silencing, and RNA interference [1,2,3,4,5,6]. 

A mirror-image of native D-nucleic acids, L-nucleic acids in a left-handed A-form (Scheme 1, left column), offers superior stability in biological environments due to its resistance to cellular nucleases [7,8]. The unique folding scaffold that L-RNA consists of is used to recognize a variety of small molecules, peptides, and proteins [9,10]. Several aptamers are now under clinical investigation [11]. Simultaneously, chimeras of L-oligonucleotide containing nanotechnology materials are employed for drug delivery by enhancing stability [12].

The left-handed Z-form double-helix structure, including Z-RNA and Z-DNA, has been shown to play an important role in multiple physiological events, including the regulation of gene expression, nucleosome positioning, and genetic instability associated with nucleic acid damage and repair (Scheme 1, right column) [13,14,15,16]. We recently suggested a relationship between this Z-form structure and several diseases, such as cancer and inflammation [3,13,14,17,18]. Double-stranded Z-RNA consists of nucleotides arranged in a pattern where the nucleobases alternate between *syn* and *anti* conformations [19]. For the typical purine/pyrimidine repeats, cytidine residues adopt the *anti* conformation, while guanosine residues display the *syn* conformation, and the phosphate backbone follows a zig–zag course in a discontinuous manner [20]. Some chemical modifications favor the *syn* conformation of guanosine, leading to a stabilized Z form [21]. We have demonstrated that trifluoromethyl introduction at the C8 position of guanosine, with the lowest energetic level, allowed guanosine to adopt the *syn* conformation to stabilize the Z form [22].

Tremendous efforts have been devoted to identifying small molecules for interaction with the Z form. For instance, metal molecules recognize Z-form DNA structures, and our previous report showed that (P)-Helicene displays chiral selection in binding to Z-DNA [23]. We recently identified a small molecule, curaxin CBL0137, which promotes the Z-form transition of DNA and RNA in the nucleus [17]. This transition activates protein Z-DNA-binding protein 1 (ZBP1)-mediated apoptosis and necroptosis, contributing to its antitumor effects. 

While Z-form nucleic acid duplexes have been widely studied, all reported structures consistently adopt a left-handed helix. None of the studies have reported on a right-handed Z-form RNA structure and its interaction with ligands. This gap in our understanding motivated us to explore whether a mirror-image, right-handed Z-form nucleic acid could exist and what unique topological features it might present. Such a discovery holds the potential to reveal new biological and medical functions, especially in understanding how nucleic acid molecules fold into complex, high-order structures and participate in vital processes and pathogenesis. Furthermore, studying the interactions between right-handed Z-form RNA and ligands could facilitate the development of novel nucleic acid-based nanomaterials, which have promising applications in drug delivery and targeted therapies. Herein, we present the first NMR structure of right-handed Z-form RNA in solution and investigate its interaction with the curaxin ligand, CBL0137.

## 2. Results

### 2.1. Synthesis of Right-Handed Z-Form RNA and Characterization by CD and ^19^F NMR

**Figure 1:**
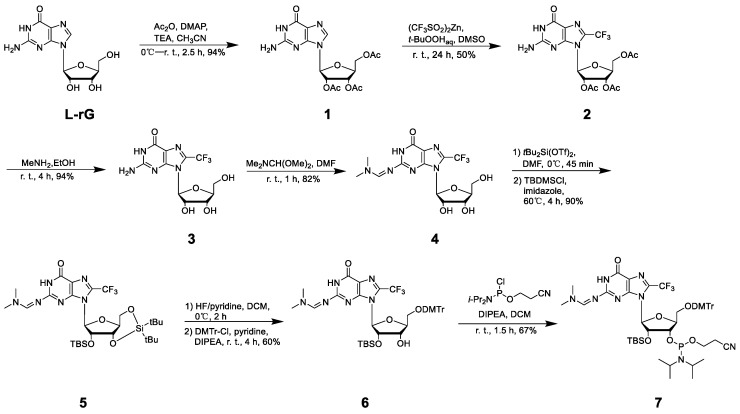
Synthetic scheme of ^F^G and relative phosphoramidite compound.

**Figure 2:**
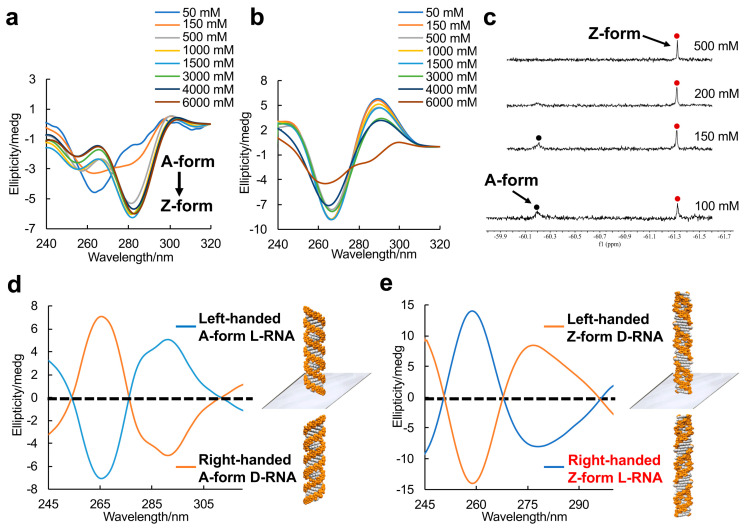
Enantiomers of A-form and Z-form RNA duplex structures. (**a**) CD spectra of the A–Z transition of L-RNA r(CGC^F^GCG)_2_ at various NaClO_4_ concentrations at 10 °C. A negative Cotton effect appears around 285 nm with increasing NaClO_4_ concentration. (**b**) CD spectra of L-RNA r(CGCGCG)_2_ at various NaClO_4_ concentrations at 10 °C. (**c**) ^19^F NMR spectra of L-RNA r(CGC^F^GCG)_2_ in 5 mM Na-PO_4_ buffer (pH 7.0) and various NaClO_4_ concentrations. Red and black spots indicated Z-form and A-form RNA, respectively. (**d**) CD spectra of A-form RNA mirror enantiomers: left-handed L-RNA r(CGCGCG)_2_ and right-handed D-RNA r(CGCGCG)_2_ in presence of 5 mM Na-PO_4_ buffer (pH 7.0) and 1.5 M NaClO_4_ at 10 °C. (**e**) CD spectra of Z-form RNA mirror enantiomers: left-handed D-RNA r(CGC^F^GCG)_2_ and right-handed L-RNA r(CGC^F^GCG)_2_ (The words in red color label highlights the typical structure which is major studying target in this study) in presence of 5 mM Na-PO_4_ buffer (pH 7.0) and 1.5 M NaClO_4_ at 10 °C.

To uncover the structural features of right-handed Z-form RNA, we designed and synthesized a novel 8-^F^G-modified L-guanosine analog (^F^G), in which a trifluoromethyl (CF_3_) group is introduced into the C8 position of L-guanosine according to a previously reported method, and incorporated it into an RNA sequence L-r(CGC^F^GCG)_2_ (Figure 1 and Supplemental Data S1–S20) [24]. As reported in our previous research, a CF_3_ group at the C8 position greatly stabilizes the Z-form nucleic acids and acts as a ^19^F sensor, which can be used to study DNA and RNA structures using ^19^F NMR [22]. We used circular dichroism spectroscopy (CD) to monitor the conformational state at various NaClO_4_ concentrations. We observed that L-r(CGC^F^GCG)_2_ greatly stabilized the Z-RNA, showing the A–Z transition in the presence of only 50 mM NaClO_4_ (Figure 2a), while the transition requires 6000 mM NaClO_4_ for unmodified L-r(CGCGCG)_2_ (Figure 2b). This result is completely consistent with ^19^F NMR, which has been successfully used to distinguish RNA structures in our previous studies (Figure 2c) [24]. In ^19^F NMR, a strong-intensity peak appeared as a Z form (−61.32 ppm) accompanied by a small peak (−60.20 ppm) at a NaClO_4_ concentration of 100 mM, indicating the major Z-form RNA and a minor A-form RNA. With increasing NaClO_4_ concentrations, only the peak of the Z-form RNA remained with a strong intensity, whereas the peak of the A-form completely disappeared.

CD was performed to distinguish the RNA mirror enantiomer. The right-handed A-form D-r(CGCGCG)_2_ exhibited a negative band around 290 nm and a positive band at 266 nm, which are typical A-form signals (Figure 2d). A left-handed A-form L-r(CGCGCG)_2_ as the enantiomer was completely antithetical. Meanwhile, the right-handed Z-form RNA L-r(CGC^F^GCG)_2_ and the left-handed Z-form RNA D-r(CGC^F^GCG)_2_ stabilized by CF_3_-modified D-guanosine showed absolutely mirror-imaged spectral signals (Figure 2e).

### 2.2. NMR Solution Structure of Right-Handed Z-Form RNA

**Figure 3:**
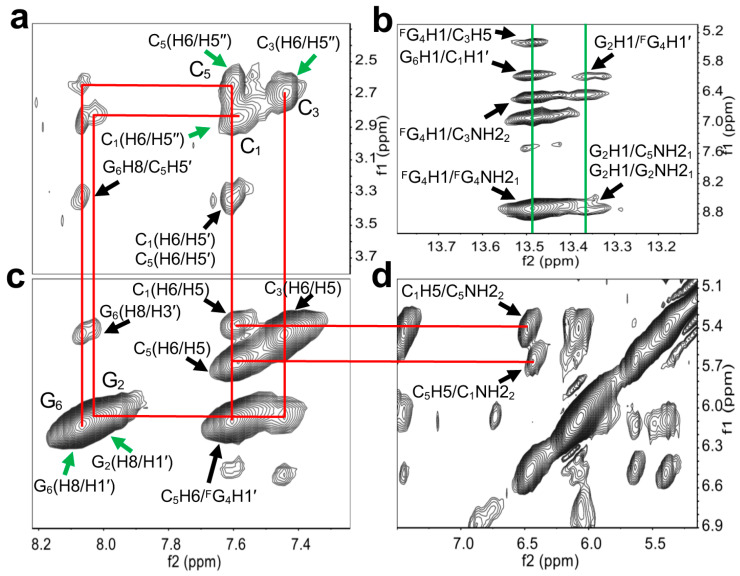
Structural determination of right-handed Z-form RNA r(C_1_G_2_C_3_^F^G_4_C_5_G_6_)_2_. (**a**,**c**,**d**) show wide-ranging connectivity paths (red lines) [C_1_(H6/H5′′)-G_2_(H8/H1′)-C_3_(H6/H5′′), C_5_(H6/H5′′)-G_6_(H8/H1′)] in the RNA strand of right-handed Z helices in a contour plot of the 2D-NOESY spectrum. (**a**) C_1_(H6/H5′′), C_3_(H6/H5′′), and C_5_(H6/H5′′) were found to upshift 2.85, 2.70, and 2.66 ppm (green arrows). Strong intra-residue cross-peaks of C_1_(H6/H5′′), C_3_(H6/H5′′), and C_5_(H6/H5′′) were observed. Additional anomeric–aromatic proton interactions were assigned as C_1_(H6/H5′), C_5_(H6/H5′), and G_6_H8/C_5_H5′, as marked by black arrows. (**b**) The cross-peaks of the imino proton of G_2_ and amino proton of C_5_, as well as the imino proton of ^F^G_4_ and the amino proton of C_3_ associated with G_6_H1/C_1_H1′, ^F^G_4_H1/C_3_H5, and G_2_H1/^F^G_4_H1′ were observed (green lines). Intraresidue NOE cross-peaks of the imino and amino protons of G_2_ and ^F^G_4_ are shown. (**c**) Strong H8–H1′ cross-peaks were observed (green arrows). C_1_(H6/H5), C_3_(H6/H5), C_5_(H6/H5), G_6_(H8/H3′), and C_5_H6/^F^G_4_H1′ are labeled by black arrows. (**d**) The Z-RNA structure-specific cross-peaks were observed between the C_5_ amino proton and C_1_H5, as well as between the C_1_ amino proton and C_5_H5 from inter-stranded interactions. These signals are linked to C_1_(H6/H5) and C_5_(H6/H5) in (**c**) by red lines.

To reveal the detailed structure of right-handed Z-form RNA, we performed 2D NMR experiments (Figure 3), in which the condition with 2 M NaClO_4_ was used to ensure complete deletion of NOEs from other structures. A complete list of ^1^H chemical shifts is shown in Supplemental Table S1. The NOE-restrained refinement provided an unequivocal demonstration that the structure of L-RNA r(CGC^F^GCG)_2_ is Z-RNA. Sequential assignments of C_1_ to C_3_ and C_5_ to G_6_ for the Z-form can complete the pathway: C_1_(H6/H5′′)-G_2_(H8/H1′)-C_3_(H6/H5′′), C_5_(H6/H5′′)-G_6_(H8/H1′) (Figure 3a,c,d), indicating a sequence-specific connectivity for right-handed helices. All H5′′ of cytidine were found to shift upfield by 2.85 (C_1_), 2.70 (C_3_), and 2.66 (C_5_) ppm; these alterations only could be observed in the Z-form nucleic acid duplex (Figure 3a). The clear cross-peaks of the imino proton of ^F^G_4_ (~13.48 ppm) and G_2_ (~13.36 ppm) with the amino proton of C_3_ (~6.49 ppm) and C_5_ (~8.74 ppm) collectively suggested Watson–Crick base pairs (Figure 3b). Moreover, the H8/H1′ cross-peak region of the 2D NOESY spectrum showed only strong intranucleotide G_2_(H8/H1′) and G_6_(H8/H1′) cross-peaks (Figure 3c), which indicated the *syn* conformation of rG residues. Strong intra-residue cross peaks of C_1_(H6/H5′′), C_3_(H6/H5′′), and C_5_(H6/H5′′) indicate *anti* conformation of all cytidines in the Z form (Figure 3a). We note that the C_1_H5 proton has cross-peaks to the C_5_ amino proton (C_1_H5/C_5_NH2_2_), and the C_5_H5 proton has cross-peaks to the C_1_ amino proton (C_5_H5/C_1_NH2_2_) (Figure 3d). Such cross-peaks can only happen between the two interstrand cytosines between C_1_ in one strand and C_5_ in another strand of the Z-RNA because of their specific base pair stacking pattern. Additionally, the NOE G_2_H3′/G_2_H4′ was observed and indicated that rG residues preserve the C3′-*endo* sugar pucker while those signals, including C_1_H1′/C_1_H2′, C_3_H1′/C_3_H2′, and C_5_H1′/C_5_H2′, demonstrated rC residues in the C2′-*endo* sugar pucker (Supplemental Figure S1).

**Figure 4:**
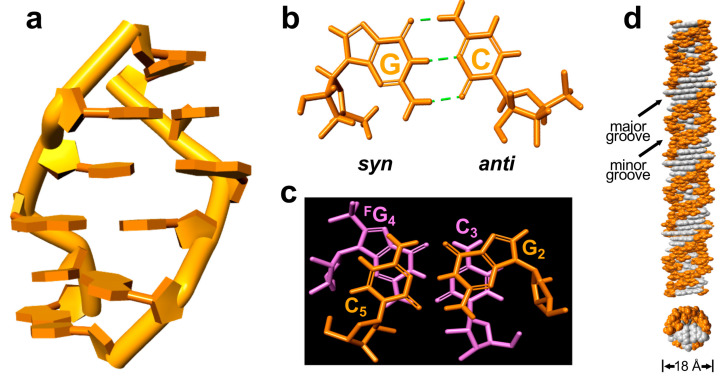
The structural model of right-handed Z-form RNA r(C_1_G_2_C_3_^F^G_4_C_5_G_6_)_2_. (**a**) Structural right-handed Z-form RNA r(CGC^F^GCG)_2_ viewed from the major groove. (**b**) rG with *syn*conformation and rC with *anti*-conformation form Watson–Crick base pairs through three hydrogen bonds in Z-form RNA. (**c**) The stacking pattern within the GpC step in Z-RNA as viewed along the helix z-axis, showing only intra- but not inter-strand stacking. (**d**) An extended model of the right-handed Z-form RNA helix from Z-form RNA r(CGC^F^GCG)_2_ visualized in side (up) and top (bottom) views to show their base pair locations and groove architecture, in which white represent base pairs and orange indicates phosphate–ribose backbones. The duplex diameter is shown, and the major or minor groove is indicated.

The structural model of L-r(CGC^F^GCG)_2_ is constructed based on the reported Z-form structure and NOE-constrained refining method (Supplemental Tables S2 and S3). The molecular dynamics simulation was carried out in BIOVIA Discovery Studio 4.5 (Accelrys, San Diego, CA, USA) through a standard dynamic cascade with some modifications. The lowest energy conformation was selected as shown in Figure 4a and Supplemental Figure S2. The Watson–Crick scheme of base pairing is observed throughout the duplex, in which rG residues are characterized by a *syn* conformation in C3′-*endo* and rC with an *anti* conformation in C2′-*endo* (Figure 4b and Supplemental Figures S3 and S4). For both the CpG and GpC steps, base stacking interactions are observed but evidently with no inter-strand base stacking (Figure 4c and Supplemental Figure S5); these results were in high agreement with the landmark of Z helices.

To further clearly observe the right-handed Z-form RNA, we generated the model of a full-turn Z-RNA helix by excluding both terminal base pairs of the 6-mer structure (Figure 4d). The right-handed RNA helix has the well-marked zig–zag shape of the phosphorsugar backbone and 2′-OH groups of rG exposed to the outer helix surface (Figure 4d and Supplemental Figure S6). This might be considered a result of the fact that the Z-RNA duplex structure requires higher salt concentration compared to Z-DNA. Another feature of the RNA helix is that both grooves are well defined, possessing a very engraved, deep and much narrower minor groove, and a shallow major groove, which is the same as its mirror image left-handed Z-form D-RNA (Supplemental Figure S6).

### 2.3. Complex Structure of Right-Handed Z-Form RNA and Ligand CBL0137

**Figure 5:**
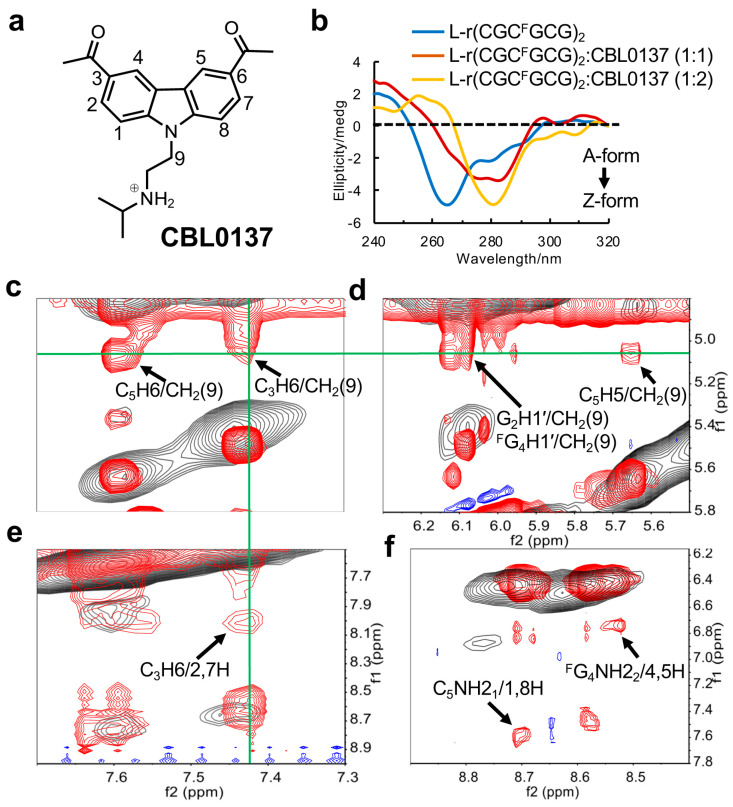
Study of the CBL0137 ligand binding to Z-form RNA. (**a**) The structure of CBL0137, comprising a carbazole moiety with a positive charged N-side chain. (**b**) The CD titration of Z-form RNA r(CGC^F^GCG)_2_ with increasing concentrations of CBL0137 at 10 °C in 5 mM Na-PO_4_ buffer (pH 7.0). The ratio of CBL0137 to RNA is indicated at the top. (c-f) The overlay of NOE spectra. The signals in red and blue from the complex of CBL0137 and RNA. The signals in black from Z-form RNA as a control. (**c**–**e**) display strong intermolecular NOEs between RNA and CBL0137. C_5_H6/CH_2_(9), C_3_H6/CH_2_(9), G_2_H1′/CH_2_(9), ^F^G_4_H1′/CH_2_(9), C_5_H5/CH_2_(9), and C_3_H6/2,7H are marked by black arrows. The signals derived from CH_2_(9) or C_3_H6 are connected by green lines. (**f**) The black arrows indicate the NOEs between amino protons C_5_NH2_1_ and the aromatic protons (1H, 8H) of CBL0137, and the NOEs between amino protons ^F^G_4_NH2_2_ and the aromatic protons (4H, 5H) of CBL0137.

We have identified a small molecule, curaxin CBL0137 (Figure 5a), which is capable of inducing Z-form formation to activate ZBP1-mediated cell death. This encouraged us to explore its binding affinity with the right-handed Z-form RNA model of right-r(CGC^F^GCG)_2_. In CD, we observed the appearance of a negative band around 280 nm with the addition of equimolar (1:1) amounts of CBL0137 (Figure 5b). This effect was even more pronounced at a higher ratio (2:1) of CBL0137 to L-r(CGC^F^GCG)_2_. These results suggest that CBL0137 effectively converts the A-RNA conformation to Z-RNA.

Furthermore, to resolve the complex structure, 1D proton NMR experiments were constructed. As an increasing amount of CBL0137 was titrated into RNA, we observed the emergence of a new set of peaks for the complex after the titration (Supplemental Figure S7). This indicated that the binding of CBL0137 to RNA can be observed in a slow exchange regime on the NMR timescale. The complete lists of ^1^H chemical shifts of complex CBL0137 and RNA are shown in Supplemental Table S4–S6. As the NMR signals preferred to be stable after the addition of double concentrations of CBL0137 into RNA, we deduced that only a dominant conformation of the 1:2 RNA–ligand complex was present. This is consistent with observations from the CD titration experiments, which showed a clear inflection point around 1:2 for the complex formation (Supplemental Figure S8). Moreover, we found that the addition of the ligand induced only a single peak of phosphate in C_3_ of the GpC step with lower intensity than the ^31^P NMR spectrum (Supplemental Figure S9), as well as a resonance shift of ^F^G_4_ in ^19^F NMR (Supplemental Figure S10).

The overlay of 2D spectra derived from the complex of CBL0137 and RNA allowed us to obtain more precise NOE interactions between the RNA and ligand (Figure 5c–f). For example, a series of cross-peaks showed the strong intermolecular interactions between CH_2_(9) in the ligand and aromatic protons such as C_5_H6, C_3_H6, and C_5_H5, as well as the sugar G_2_H1′ and ^F^G_4_H1′ protons of the RNA. Similarly, the NOEs between the aromatic protons of CBL0137 and the amino protons or aromatic protons of the RNA were detected. These results suggested that the ligand may insert into G_2_:C_5_ and C_3_:^F^G_4_ base pairs via the stacking effect. 

**Figure 6:**
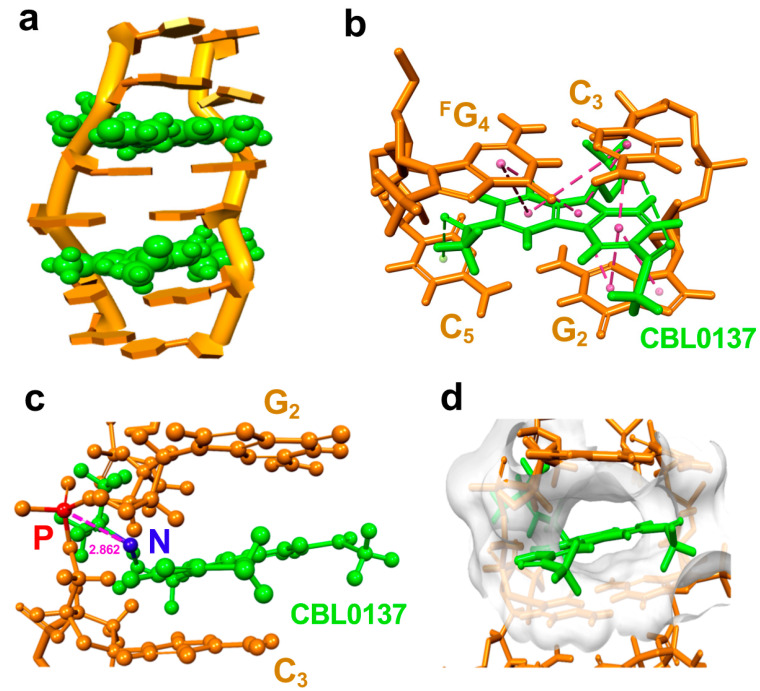
The molecular dynamic model of CBL0137 ligand binding to Z-form right-hand RNA. (**a**) Solution structure of the CBL0137-RNA complex with cartoon representation. CBL0137 (in green CPK representation) symmetrically binding with Z-form RNA (orange) in a 2:1 molecular ratio. (**b**) An expanded view of CBL0137 (in green stick representation) and base pairs indicates the π-π stacking formation between the carbazole moiety and base pairs of G_2_:C_5_ and C_3_:^F^G_4_ as pink dashed lines connected by each aromatic ring’s center. (**c**) A side view showing the close proximity between the cationic N-side chain (shown as a blue sphere) of CBL0137 and the anionic phosphate group (shown as a red sphere) of C_3_ (~2.862 Å in distance). (**d**) The solvent hydrophobicity representation of the surface of RNA (white) shows CBL0137 (in green stick representation) positioned in the cleft pocket.

The solution structure of the CBL0137-RNA complex was computed using NMR restraints (Figure 6a and Supplemental Figure S11). Two ligands were observed to symmetrically insert into the G_2_:C_5_ and C_3_:^F^G_4_ base pairs at the GpC step through π stacking. An expanded view shows clearly the π-π stacking interactions between the carbazole moiety of CBL0137 and the base pairs (Figure 6b and Supplemental Figure S12). The cationic side chain of the ligand approached the phosphate backbone of C_3_ through intense electrostatic interactions between the positively charged nitrogen atom of the ligand and the negatively charged phosphate backbone of C_3_ (Figure 6c), while the acetyl groups at the positions 3 and 7 of carbazole protruded into the major groove (Supplemental Figure S13). This is in agreement with the observed NOE cross-peak between the phosphate of C_3_ and hydrogen of CBL0137 isopropyl from ^31^P–^1^H HOESY (heteronuclear Overhauser effect spectroscopy) NMR (Supplemental Figure S14).

We noted that π-π stacking between the planar carbazole moiety and base pairs of RNA produces a hydrophobic cavity as a cleft pocket in RNA (Figure 6d and Supplemental Figure S15). This suggests that the CBL0137 binding to Z-form RNA may result from a preference that Z-form RNA provides such a cleft pocket, whereas A-form RNA lacks the space for CBL0137 binding. This is consistent with the higher binding constant K = 1.63 × 10^5^ M^−1^ of CBL0137 and the right-handed Z-form RNA r(CGC^F^GCG)_2_ complex compared to that of K = 4.31 × 10^4^ M^−1^ of CBL0137 and the right-handed A-form RNA r(CGCGCG)_2_ complex (Supplemental Figure S16).

## 3. Discussion

The present data clearly show the right-handed Z-form L-RNA are the exact mirror images of left-handed helix Z-form D-RNA; except for chirality, both possess the same conformation. Such a fine structure provides an essential piece of information in the higher order structures of RNAs, completing the puzzle of mirror images of RNA A-form and Z-form enantiomers. 

Previous studies revealed that Z-RNA plays critical roles in antiviral defense and cancer therapy, which has generated significant research interest. Additionally, L-nucleic acids are highly resistant to enzymatic degradation, making them ideal candidates for nanomaterial applications in biosensing and drug delivery. These results consistently demonstrate that Z-form L-RNA possesses potentially applicable values in the biological and medical fields. Moreover, the incorporated 8-^F^G-modified L-guanosine could serve as a sensitive tool for ^19^F NMR studies, enabling the observation and investigation of the biological functions of Z-form L-RNA within cells.

We demonstrated that curaxin CBL0137 interacts with right-handed Z-form RNA predominantly through π stacking and electrostatic interactions. The conjugated aromatic ring of CBL0137 adopts a centrosymmetric orientation, facilitating π-π stacking interactions with the base pairs of the upper G2 and lower C3. However, the N-side chain of CBL0137 does not localize symmetrically but shifts towards the phosphate backbone of the C3 residue. This shift enables strong electrostatic interactions between the positively charged nitrogen of CBL0137 and the negatively charged phosphate backbone of C3 [25]. From the resolved structure of the complex, we infer that enhancing the binding affinity or selectivity of small molecules towards specific Z-form RNA structures can be achieved by optimizing the steric properties of the planar stacking and the cationic properties of the side chain. 

Previous studies indicated that the Z-form DNA comprising 2′-deoxy-L-ribose adopts a right-hand helix and could bind with small molecules [26,27]. These results, corroborated with the findings in this study, are perfectly in line with our recent report, which showed that CBL0137 triggers Z-form formation and contributes to its antitumor effects. Advanced NMR studies on the resulting complex of left-handed helix Z-form D-RNA and CBL0137 and the implications of their formation are currently underway.

## 4. Materials and Methods

### 4.1. RNA Sample Preparation

By using an automatic solid-phase phosphoramidite chemistry and DNA/RNA synthesizer, the d-, l-, and trifluoromethyl-labeled RNAs were synthesized at a ratio of 1.0 µmol. Following the RNAs being cleaved from the column and deprotected by using Ammonium Hydroxide/40% aqueous methylamine 1:1 *v/v* (AMA) at room temperature for 20 min and at 65 °C for 10 min, respectively. Tert-butyldimethylsilyl (TBDMS) protections were removed by treatment with triethylamine trihydrofluoride followed by filtration through an ion exchange cartridge. The oligomers were further purified by high-performance liquid chromatography (HPLC) in a linear gradient of 50 mM ammonium formate in 1:1 acetonitrile/H_2_O and 50 mM ammonium formate in H_2_O. The oligomers were desalted through a NAP 10 column (disposable column, GE Healthcare) and identified by matrix-assisted laser desorption/ionization time-of-flight mass spectrometry (MALDI-TOF-MS) on an Autoflex III smart beam mass spectrometer (negative mode) (Supplemental Data S21–S24). 

### 4.2. Circular Dichroism 

CD experiments were performed by using a JASCO model J-820 CD spectrophotometer (JASCO Corporation, Tokyo, Japan). The RNA samples were prepared at a 10 μM concentration in the presence of different concentrations of NaClO_4_, and 5 mM Na-PO_4_ buffer (pH 7.0). For the CBL0137 and RNA binding assay, RNAs were prepared at 10 µM in 5 mM NaPO_4_ buffer (pH 7.0). CBL0137 was added into the RNA solution and kept at room temperature for 30 min before measurement. Titrating concentrations for ligands were prepared at [RNA duplex]/[CBL0137] ratios of 1:0, 1:1, and 1:2 in the presence of a fixed concentration (5 μM) of the duplex RNA. The binding constant K was calculated by analyzing CD data at a fixed wavelength versus ligand concentrations according to our previous study [28].

### 4.3. ^1^H NMR Experiments

NMR data were recorded on a BRUKER AVANCE 400 and 600 MHz spectrometer (Bruker, Billerica, MA, USA). For spectra recorded in 90% H_2_O/10% D_2_O water, the signal was suppressed using the 3-9-19 WATER-GATE (water suppression by gradient-tailored excitation) pulse sequence or excitation sculpting with a gradient pulse. The data were processed with TopSpin 3.0 (Bruker BioSpin Gmbh) software and analyzed with MestReNova software (Mestrelab Research, USC, ES). For 1D NMR measurement (^1^H and ^31^P spectrum), RNA samples of a concentration of 0.3 or 2 mM were dissolved in 150 µL of the designed solution containing 10% D_2_O, 2 M NaClO_4_ and 10 mM Na-PO_4_ buffer (pH 7.0). Two-dimensional NOESY spectra in 90% H_2_O/10% D_2_O were collected from 512 scans with a 400 ms mixing time at 20 °C. On average, 2048 complex points and 512 FIDs (free induction decays) were collected within the spectral width of 14,097 Hz. The sample solutions were as follows: 2 mM RNA were dissolved in 150 µL of designed solution containing 10% D_2_O, 2 M NaClO_4_, and 10 mM Na-PO_4_ buffer (pH 7.0). Heteronuclear 2D NOESY spectra (^1^H–^31^P) were carried out in an identical solution containing 10% D_2_O, 2 M NaClO_4_, and 10 mM Na-PO_4_ buffer (pH 7.0), in which the chemical shift of phosphate from Na-PO_4_ was referred to as the internal standard at 0 ppm. The samples were prepared by heating the oligonucleotides at 85 °C for 3 min and gradually cooling them to room temperature. To study the RNA duplex binding with the CBL0137 ligand, the samples were prepared by the denaturing and annealing of RNA and followed by incubation at 4 °C overnight before the addition of the ligand. Conditions: 0.15 or 1 mM RNA duplex with 0.3 or 2 mM CBL0137 dissolved in the solution of 2 M NaClO_4_ and 10 mM Na-PO_4_ buffer (pH 7.0) containing 10% D_2_O.

### 4.4. ^19^F NMR Experiments

For ^19^F NMR measurement, RNA samples of a concentration of 100 μM were dissolved in 150 μL of the designed solution containing 10% D_2_O, in the presence of different concentrations of NaClO_4_ and 5 mM Na-PO_4_ buffer (pH 7.0). The samples were prepared by heating the ^19^F-labeled oligonucleotides at 90 °C for 3 min and gradually cooling them to room temperature. The ^19^F NMR spectrum was measured on a Bruker AVANCE 400 MHz spectrometer (Bruker, Billerica, MA, USA) at a frequency of 376.05 MHz and referenced to the internal standard CF_3_COOH (–75.66 ppm). The experimental parameters are recorded as follows: spectral width 89.3 kHz, ^19^F excitation pulse 15.0 μs, relaxation delay 1.5 s, acquisition time 0.73 s, scan numbers 1000, and temperature 10 °C.

### 4.5. Structural Determination

All assigned NOESY cross peaks were classified as strong (1.8–3.0 Å), medium (3.0–3.7 Å), weak (3.7–5.5 Å), and very weak (5.5–7.5 Å) inter-proton distance restraints based on the intensity of NOESY. The average of cytosine H5-H6 proton distances was used as a reference (2.42 Å). Distance restraints for the hydrogen bonding in each Watson–Crick base pair were 1.8–3.7 Å. The force constant of hydrogen bonds and NOE restraints were kept between 5 and 50 kcal mol^−1^ Å^−2^ throughout the computation. Then, molecular dynamics simulations were performed by the structural solvation and standard dynamics cascade in BIOVIA Discovery Studio 4.5 with modifications. Generally, the structure was heated from 50 K to 300 K over 4 ps and equilibration at 300 K with 100 ps simulation time. The save results interval in the production step was 2 ps during 100 ps simulation time at 300 K. The 10 best conformations generated by simulation were further energy minimized until the gradient of energy was less than 0.1 kcal mol^−1^. The conformation with the lowest energy was selected as the final presentation. The all-atom RMSD was calculated using Z-form L-RNA in superimposition.

### 4.6. Molecular Modeling

We manually prepared the initial model using L-ribonucleosides, which are the mirror images of D-ribonucleosides by using the L-RNA model (PDB: 1R3O) with A-form structure served as the template [29]. Next, the structure was modified as the known structural features of Z-form D-RNA and D-DNA with the *syn* conformation of guanosine and *anti* conformation of cytidine, which are consistent with the NMR results in this study. All of the above molecular model constructions were accomplished by BIOVIA Discovery Studio 4.5.

### 4.7. Molecular Docking Simulation

The molecular docking calculations were performed by using the BIOVIA Discovery Studio 4.5. All assigned NOESY cross-peaks in the Z-form L-RNA duplex–CBL0137 complex were used to determine the inter-proton distance restraints. The NOE peaks of H5-H6 from the cytosine bases were used for calibration of the distance measurements. The structure of CBL0137 was optimized before use as a ligand in the binding assay. The Z-form L-RNA duplex was collected from the former NMR structure in the solution in this study. All possible docking sites were provided by the BIOVIA Discovery Studio 4.5 based on the calculation results and were further selected reasonably according to their NOEs intensities. The docking experiment was implemented and provided at least five conformations, in which the proper one was used for the subsequent standard dynamics cascade and energy minimization processes. A total of five conformations were generated and the conformation with lowest energy was selected for the final presentation. The all-atom RMSDs were calculated using the Z-form L-RNA-CBL0137 complex in superimposition.

## 5. Conclusions

In summary, we described a right-handed Z-form L-RNA, and demonstrated its binding with the CBL0137 ligand at a high resolution. These results indicate that the new nucleic acid structure might produce applicable biofunctions.

## Data Availability

The raw data supporting the conclusions of this article will be made available by the authors upon request.

## Supplementary Materials

The following supporting information can be downloaded at: https://www.mdpi.com/article/10.3390/molecules29204900/s1, Figure S1: Anomeric regions of Z-form RNA in 2D NOESY; Figure S2: Structural model of Z-form RNA L-r(CGC^F^GCG)_2_; Figure S3: Inter-strand Watson–Crick base pairs involved in the Z-form RNA duplex; Figure S4: Stick model of Z-form RNA; Figure S5: Stacking pattern within the CpG and GpC steps in Z-form RNA as viewed along the helix z-axis; Figure S6: The refined structure of Z-form RNA L-r(C_1_G_2_C_3_^F^G_4_C_5_G_6_)_2_ is a half turn of a right-handed RNA helix; Figure S7: 1D NMR spectra of Z-form L-r(CGC^F^GCG)_2_ with CBL0137; Figure S8: Study of the CBL0137 ligand binding to Z-form RNA; Figure S9: ^31^P spectra of Z-form L-r(CGC^F^GCG)_2_/CBL0137 complex; Figure S10: ^19^F spectra of Z-form L-r(CGC^F^GCG)_2_/CBL0137 complex; Figure S11: Structural model of Z-form RNA L-r(CGC^F^GCG)_2_ and the CBL0137 complex; Figure S12: An expanded view of the RNA and CBL0137 complex models; Figure S13: Molecular model show the acetyl groups (purple, CPK presentation) at the position of 3 and 7 of carbazole (green, CPK presentation) protruding into the major groove of the Z-form duplex; Figure S14: 2D heteronuclear ^31^P-^1^H NMR spectroscopy of the Z-form RNA L-r(CGC^F^GCG)_2_ and the CBL0137 complex; Figure S15: Molecular model of Z-form L-r(CGC^F^GCG)_2_/CBL0137 complex; Figure S16: Titration data of CBL0137 and RNA derived by monitoring the wavelength at 280 nm of CD. Data S1-S20: Synthesis of 8-^F^G-modified L-guanosine phosphoramidite; Data S21–S24: MALDI-MS of oligonucleotide in this study; Table S1: ^1^H chemical shift assignments of Z-form RNA L-r(CGC^F^GCG)_2_; Table S2: Statistics for the structure determination of Z-form RNA L-r(CGC^F^GCG)_2_; Table S3: A comprehensive list of NMR distance restraints of Z-form RNA L-r(CGC^F^GCG)_2_; Table S4: ^1^H chemical shift assignments of CBL0137; Table S5: ^1^H chemical shift assignments of Z-form RNA L-r(CGC^F^GCG)_2_ in presence of CBL0137; Table S6: ^1^H chemical shift assignments of CBL0137 in the presence of Z-form RNA L-r(CGC^F^GCG)_2_.
