# Supplementary material for: Mirror-Image RNA: A Right-Handed Z-Form RNA and Its Ligand Complex
Source: Molecules. 2024 Oct 16;29(20):4900. doi: 10.3390/molecules29204900 (PMC11510240; doi:10.3390/molecules29204900)
Supplement: Supplementary file 1 [file molecules-29-04900-s001.zip › molecules-3241771-SI.pdf]

Mirror-Image RNA: A Right-Handed Z-Form RNA and Its Ligand Complex

Yi Song [1], Shiyu Wang [1] and Yan Xu [1,*]

[1]Division of Chemistry, Department of Medical Sciences, Faculty of Medicine, University of Miyazaki, 5200 Kihara, Kiyotake, Miyazaki 889-1692, Japan.

E-mail: xuyan@med.miyazaki-u.ac.jp

*Authors to whom correspondence should be addressed.

Supplemental Table S1. ^{1}H chemical shift assignments of Z-form RNA L-r(CGC^{F}GCG)$_2$[a].

Residues	H8/H6	H5	H1′	H2′	H3′	H4′	H5′	H5″	imino	amino
C_1	7.59	5.36	6.10	4.14	4.72	4.23	3.30	2.85	—	8.58<br>6.41
G_2	8.03	—	6.06	4.46	5.35	4.88	4.40	na	13.36	8.54<br>6.43
C_3	7.44	5.39	6.03	4.22	4.70	4.15	3.85	2.70	—	8.64<br>6.49
$^FG_4$	—	—	6.08	4.33	5.16	na	4.29	4.27	13.48	8.69<br>6.89
C_5	7.60	5.63	6.05	4.24	4.75	na	3.34	2.66	—	8.74<br>6.44
G_6	8.06	—	6.12	4.47	5.42	4.01	na	na	13.50	7.50<br>6.83

[a]na is "not applicable", representing ^{1}H chemical shifts could not be determined in NOESY spectrum.

Supplemental Table S2. Statistics for the structure determination of Z-form RNA L-r(CGC^{F}GCG)$_2$[a].

Structure determination by NMR restraints	
Watson–Crick pair distances	18
Planarity of base pairs	6
NOE intra-residue distances	92
NOE inter-residue distances	48
Total restraints/residue	26
Structure analysis of the 5 best conformers[a]	
Average deviation from ideal geometry	
Bond length	0.00214 ± 0.00005 Å
Bond angles	0.5273 ± 0.0036°
Number of NOE violations (>0.2 Å)	0

[a]The best conformers represent these structures adopting lowest energies following structural calculation.

Supplemental Table S3. A comprehensive list of NMR distance restraints of Z-form RNA L-$r(CGC^{F}GCG)_2$.

Constraints numer	Nucleoside name 1	Atom 1	Nucleoside name 2	Atom 2	NOEs integration value	NOEs integration value (S.D.)[a]	Constraints distance value	Constraints distance value (S.D.)[b]	Classcification
1	C_1	H6	C_1	H5’	0.41	0.03	4.11	2.43	weak (3.7-5.5 Å)
2	C_1	H6	C_1	H5”	0.55	0.05	3.54	2.39	medium (3.0-3.7 Å)
3	C_1	H6	C_1	H5	0.96	0.04	2.43	0.12	strong (1.8-3.0 Å)
4	C_1	H5	C_5	$NH2_2$	0.68	0.04	3.61	0.17	medium (3.0-3.7 Å)
5	C_1	H1’	C_1	H2’	0.73	0.06	3.20	0.32	medium (3.0-3.7 Å)
6	G_2	H8	G_2	H1’	0.92	0.03	2.44	1.36	strong (1.8-3.0 Å)
7	G_2	H1	$^{F}G_4$	H1’	0.34	0.02	6.89	1.43	very weak (5.5-7.5 Å)
8	G_2	H1	G_2	$NH2_1$	0.68	0.04	3.33	0.20	medium (3.0-3.7 Å)
9	G_2	H1	C_5	$NH2_1$	0.63	0.03	4.24	0.24	weak (3.7-5.5 Å)
10	G_2	H3’	G_2	H4’	0.79	0.06	2.95	0.68	strong (1.8-3.0 Å)
11	C_3	H6	C_3	H5”	0.65	0.04	3.23	1.26	medium (3.0-3.7 Å)
12	C_3	H6	C_3	H5	1.00[c]	0.00	2.35	0.11	strong (1.8-3.0 Å)
13	C_3	H1’	C_3	H2’	0.72	0.05	3.06	0.61	medium (3.0-3.7 Å)
14	$^{F}G_4$	H1	C_3	$NH2_2$	0.49	0.06	6.29	0.64	very weak (5.5-7.5 Å)
15	$^{F}G_4$	H1	$^{F}G_4$	$NH2_1$	0.80	0.04	3.49	0.73	medium (3.0-3.7 Å)
16	$^{F}G_4$	H1	C_3	H5	0.42	0.03	5.72	0.57	very weak (5.5-7.5 Å)
17	C_5	H6	C_5	H5’	0.34	0.02	3.30	1.31	medium (3.0-3.7 Å)
18	C_5	H6	C_5	H5”	0.46	0.02	4.55	1.39	very weak (5.5-7.5 Å)
19	C_5	H6	C_5	H5	0.95	0.03	2.47	0.12	strong (1.8-3.0 Å)
20	C_5	H6	$^{F}G_4$	H1’	0.50	0.02	5.74	1.41	very weak (5.5-7.5 Å)
21	C_5	H5	C_1	$NH2_2$	0.67	0.05	3.80	0.60	weak (3.7-5.5 Å)
22	C_5	H1’	C_5	H2’	0.76	0.04	2.91	0.57	medium (3.0-3.7 Å)
23	G_6	H8	G_6	H5’	0.34	0.02	6.96	2.76	very weak (5.5-7.5 Å)
24	G_6	H8	G_6	H3’	0.42	0.06	5.37	2.63	weak (3.7-5.5 Å)
25	G_6	H8	G_6	H1’	0.97	0.06	2.41	2.20	strong (1.8-3.0 Å)
26	G_6	H1	C_1	H1’	0.35	0.03	7.02	1.87	very weak (5.5-7.5 Å)

[a]S.D. represents standard deviations from three repetitive calculation of NOEs integration in NOESY (n = 3).

[b]S.D. represents standard deviations of atomic distances from all 10 dynamic molecular models in molecular determination (n = 10).

[c]The NOE peaks of H5-H6 from C_3 were used as calibration for the distance measurements.

Supplemental Table S4. ^{1}H chemical shift assignments of CBL0137.

protons	1,8H	2,7H	4,5H	$CH_2(9)$	CH_2	CH (isopropyl)	CH_3 (isopropyl)	CH_3CO
δ (ppm)	7.94	8.44	8.74	4.88	3.86	3.57	1.40	2.45

Supplemental Table S5. ^{1}H chemical shift assignments of Z-form RNA L-r(CGC^{F}GCG)$_2$ in presence of CBL0137[a].

Residues	H8/H6	H5	H1′	H2′	H3′	H4′	H5′	H5″	imino	amino
C_1	7.58	5.35	6.09	4.12	4.70	na	3.32	2.84	—	8.59<br>6.40
G_2	8.01	—	6.12	4.48	5.32	4.86	4.39	3.89	9.15	8.53<br>6.46
C_3	7.43	5.48	6.03	4.24	4.74	na	3.85	2.76	—	8.62<br>6.51
$^{F}G_4$	—	—	6.08	4.35	na	na	4.31	4.25	13.48	8.68<br>6.74
C_5	7.60	5.65	6.04	4.25	4.72	na	3.36	2.66	—	8.72<br>6.49
G_6	8.04	—	6.13	4.49	5.44	4.01	3.99	na	13.50	7.52<br>6.86

[a]na is “not applicable”, representing ^{1}H chemical shifts could not be determined in NOESY spectrum.

Supplemental Table S6. ^{1}H chemical shift assignments of CBL0137 in presence of Z-form RNA L-r(CGC^{F}GCG)$_2$.

protons	1,8H	2,7H	4,5H	$CH_2(9)$	CH_2	CH (isopropyl)	CH_3 (isopropyl)	CH_3CO
δ (ppm)	7.60	8.01	8.52	5.06	4.05	3.55	1.58	2.63

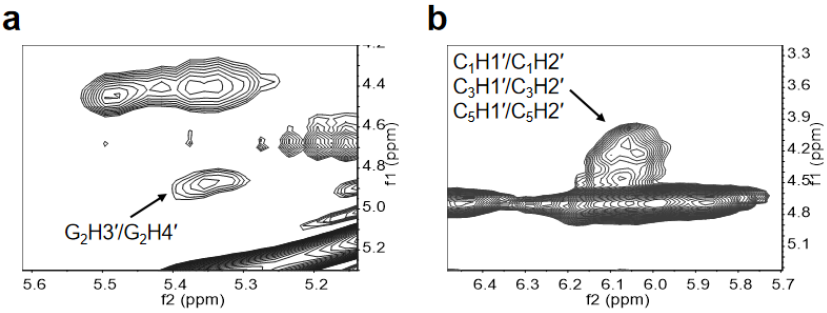

Figure S1. Anomeric regions of Z-form RNA in 2D NOESY. (a) Intranucleotide cross peaks, G_2H3'/G_2H4' indicated that G_2 residue in C3′-*endo* sugar pucker, marking by black arrow. (b) The NOEs H1′/H2′ from all three cytidines showed that all C residues in C2′-*endo* sugar pucker and marked by black arrow.

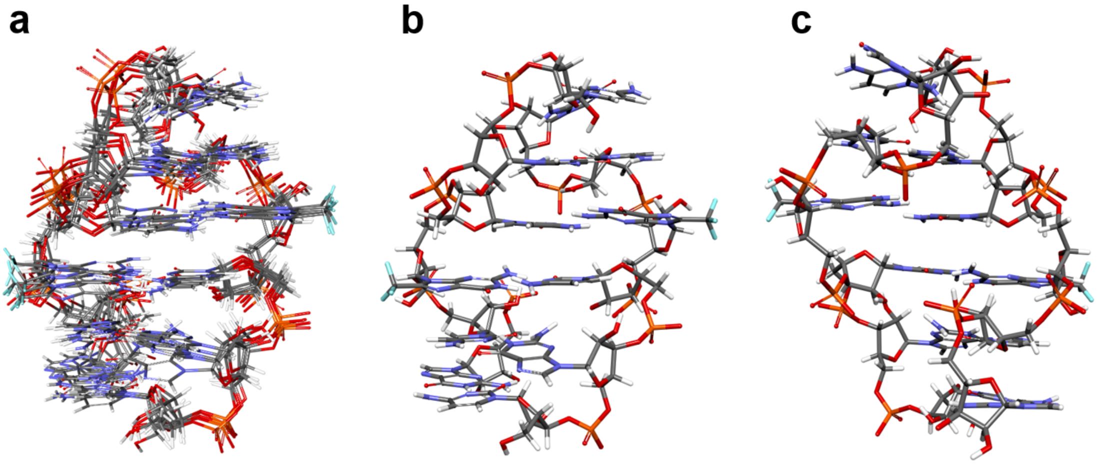

Figure S2. Structural model of Z-form RNA L-r($CGC^FGCG)_2$. (a) an ensemble of lowest energy structures. The duplex with an RMSD deviation of 0.98 Å. Stereoviews of structures viewed at major (b) and minor (c) grooves.

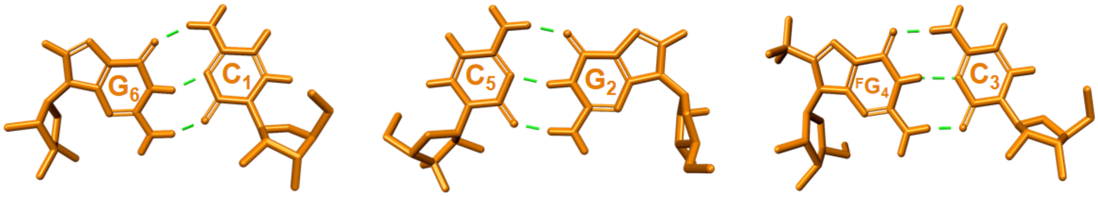

Figure S3. Inter-strand Watson-Crick base pairs involved in the Z-form RNA duplex, where all nucleosides with numbers were indicated. Green dashed lines showing three hydrogen bonds in each C:G base pair that strongly stabilize the duplex formation.

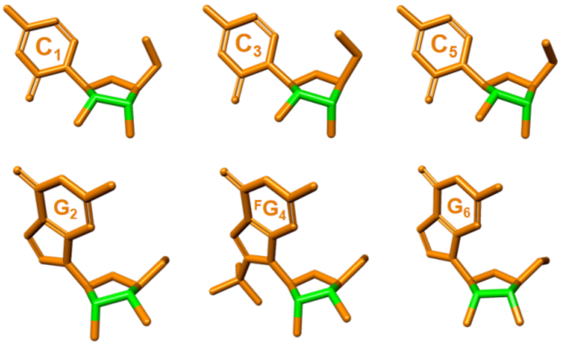

Figure S4. Stick model of Z-form RNA. All cytidines showed the remarkable C2′-*endo* sugar pucker as well as all guanosines indicated C3′-*endo* sugar pucker in the Z-form RNA duplex.

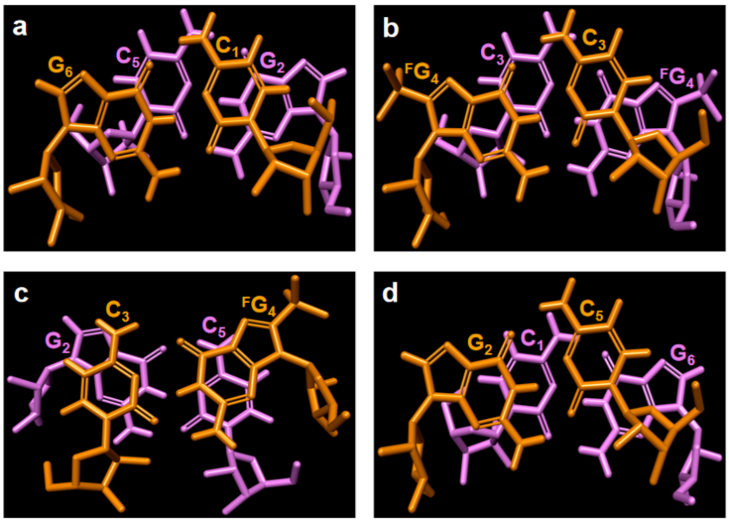

Figure S5. Stacking pattern within the CpG and GpC steps in Z-form RNA as viewed along the helix z-axis. (a) The CpG step between base pairs C_1:G_6 and G_2:C_5. (b) The CpG step between base pairs C_3:$^FG_4$ and $^FG_4$:C_3. (c) The GpC step between base pairs $^FG_4$:C_3 and C_5:G_2. (d) The CpG step between base pairs C_5:G_2 and G_6:C_1.

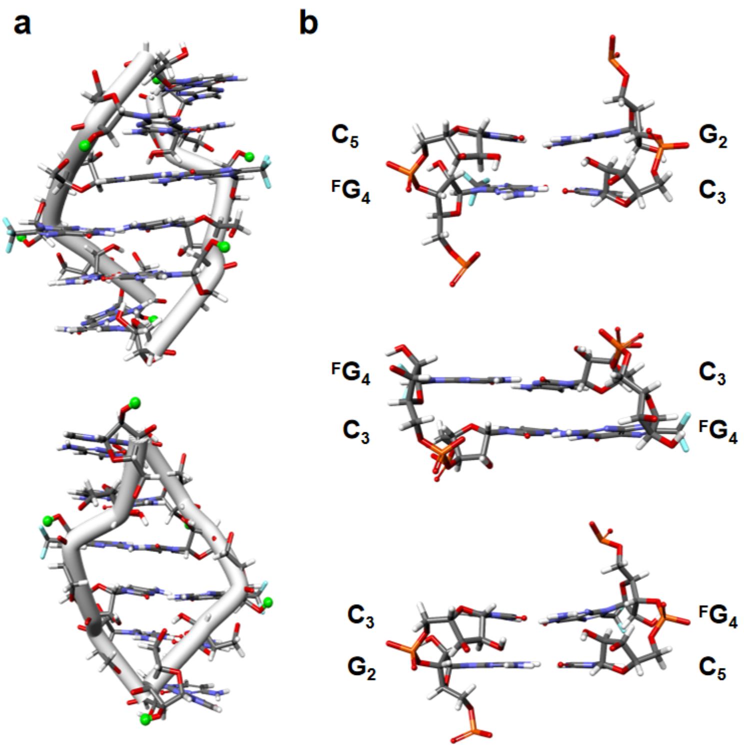

Figure S6. The refined structure of Z-form RNA L-r($C_1G_2C_3{}^FG_4C_5G_6$)$_2$ is a half turn of a right-handed RNA helix. (a) The structure of Z-form RNA as viewed from the major (top column) and minor (bottom column) groove, indicating guanosine 2′-OH groups are strongly exposed to the outer helix surface, labeled by green balls. (b) Stacking pattern within the CpG and GpC steps in Z-form RNA as viewed from the minor groove.

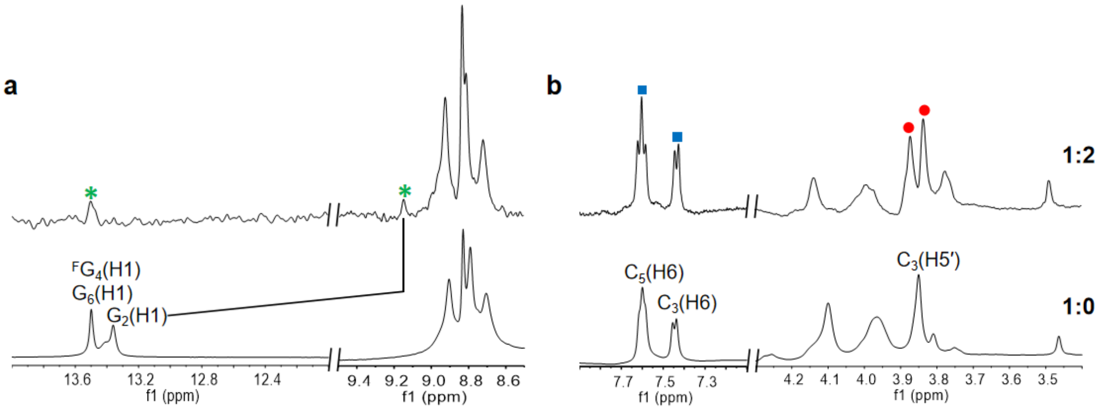

Figure S7. 1D NMR spectra of Z-form L-r(CGC^{F}GCG)$_2$ with CBL0137 at 10 °C in H_2O/D_2O (9:1). (a) Imino proton region of the 1D NMR shows G_2(H1) in upfield shift after complex formation, labeled by green marks. (b) Blue rectangles and red spheres respectively represent the splitting effects of proton peaks from C_3(H6), C_5(H6) and C_3(H5′) from [RNA duplex]/[CBL0137] complex. Condition: 2 mM RNA in 10 mM Na-PO_4 and 2 M $NaClO_4$ at pH 7.0, with the [RNA duplex]/[CBL0137] ratios at 1:0 and 1:2.

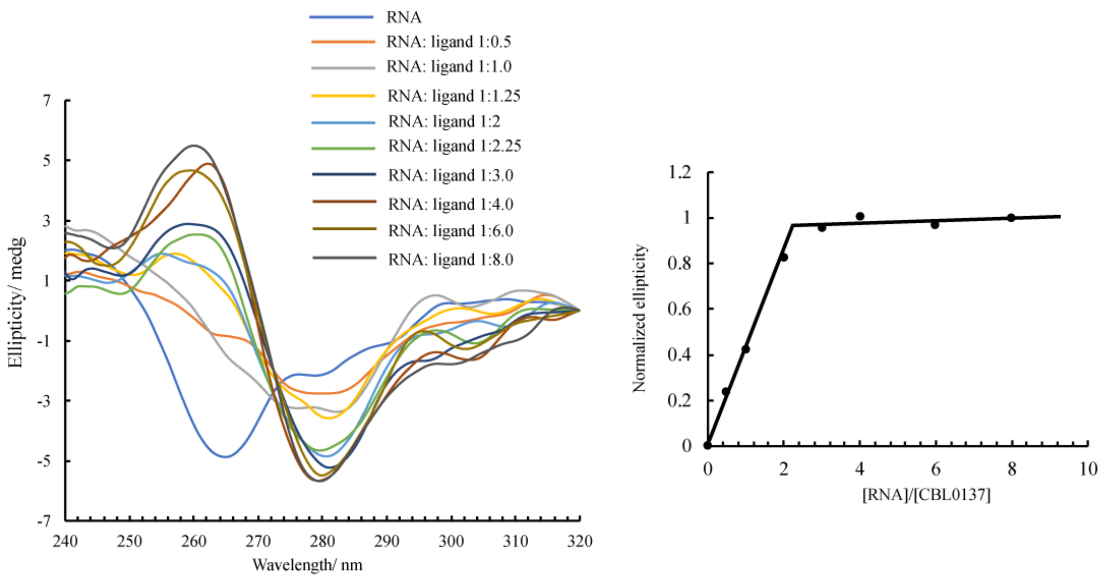

Figure S8. Study of CBL0137 ligand binding to Z-form RNA. (a) CD titration of Z-form RNA r(CGC^{F}GCG)$_2$ with increasing concentrations of CBL0137 at 10 °C in 5 mM Na-PO$_4$ buffer (pH 7.0). Ratio of CBL0137 and RNA is indicated. (b) Job plot of CD cotton effect monitored at 280 nm based on the result of CD.

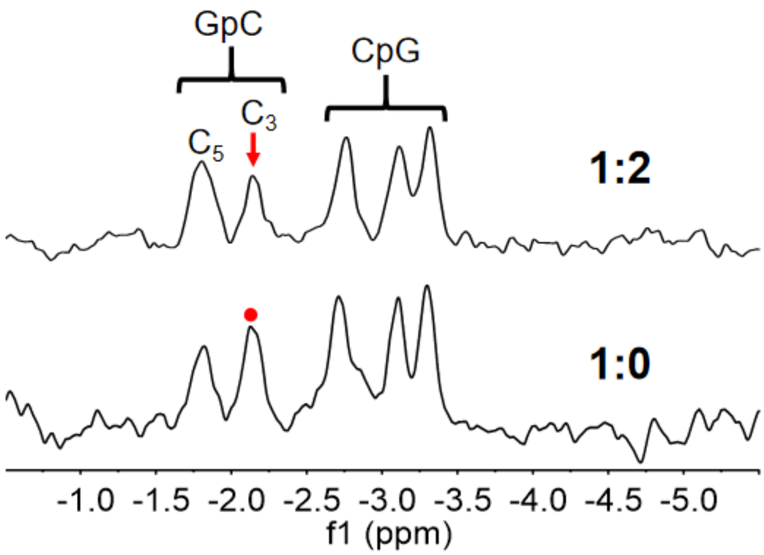

Figure S9. ^{31}P spectra of Z-form L-r(CGC^{F}GCG)$_2$/CBL0137 complex at 10 °C in H_2O/D_2O (9:1). Red ball represents the ^{31}P resonance from C$_3$ residue, showing decreased intensity after binding with CBL0137 based on the calculation of peaks integration at bottom. Condition: 0.3 mM RNA in 10 mM Na-PO$_4$ and 2 M NaClO$_4$ at pH 7.0, with different [RNA duplex]/[CBL0137] ratios, 1:0 and 1:2.

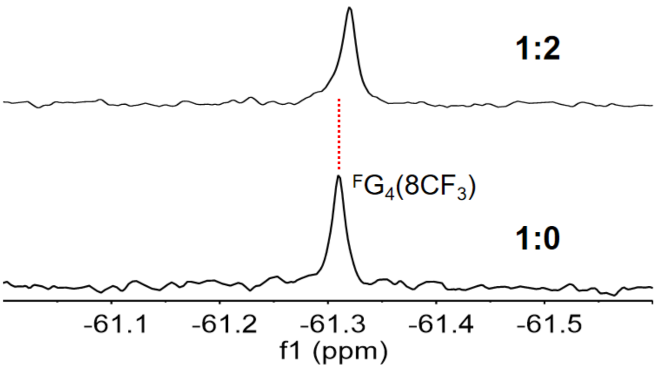

Figure S10. ^{19}F spectra of Z-form L-r(CGC^{F}GCG)$_2$/CBL0137 complex at 10 °C in H_2O/D_2O (9:1). The red dotted line indicated the ^{19}F signal from $^{F}G_4$ residue in upfield shift after binding with CBL0137. Condition: 0.3 mM RNA in 10 mM Na-PO_4 and 2 M $NaClO_4$ at pH 7.0, with different [RNA duplex]/[CBL0137] ratios, 1:0 and 1:2.

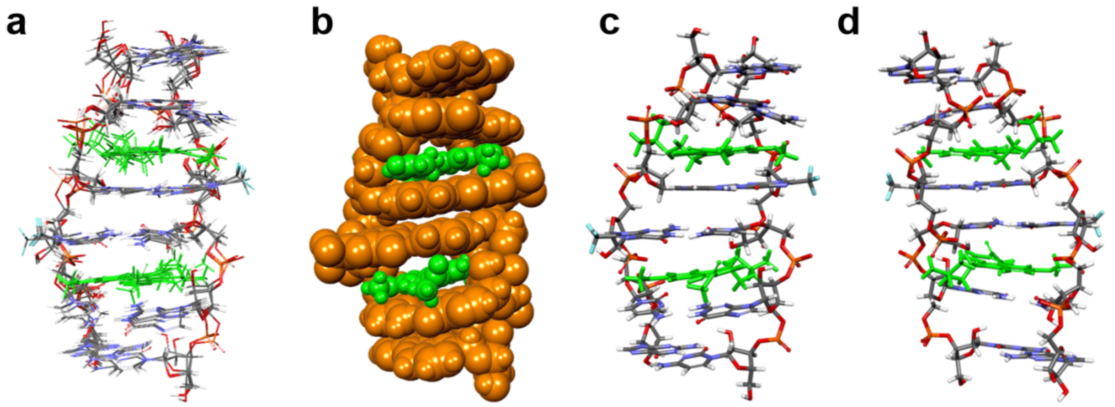

Figure S11. Structural model of Z-form RNA L-r(CGC^{F}GCG)$_2$ and CBL0137 complex. (a) Stereoviews of an ensemble of lowest energy structures. The duplex with an RMSD deviation of 0.59 Å. (b) CPK model of the CBL0137-RNA complex with cartoon representation. CBL0137 (green) symmetrically binding with Z-form RNA (orange) in 2:1 molecular ratio. Stereoviews of structures viewed at major (c) and minor (d) grooves.

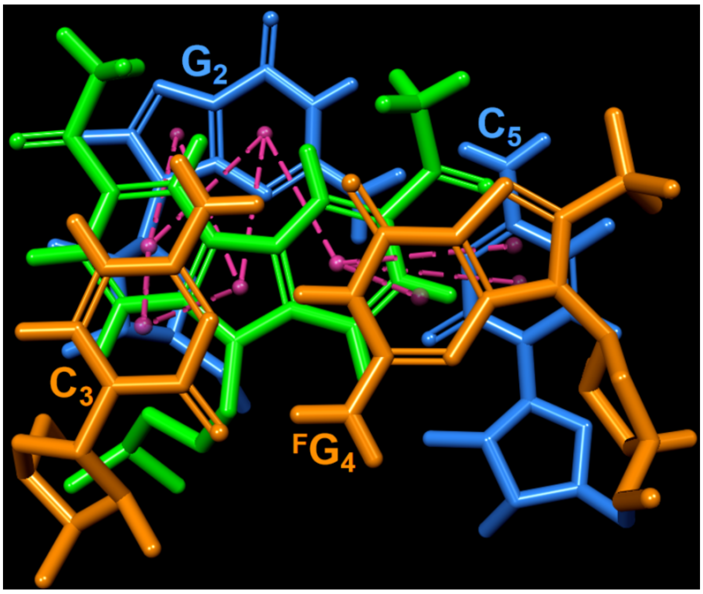

Figure S12. An expanded view of RNA and CBL0137 complex model along the helix z-axis, indicating π-π stacking conjugate formation between carbazole moiety (green) and base pairs of G_2:C_5 (blue) and C_3:$^FG_4$ (brown) in duplex as pink dashed lines connected by each aromatic rings center.

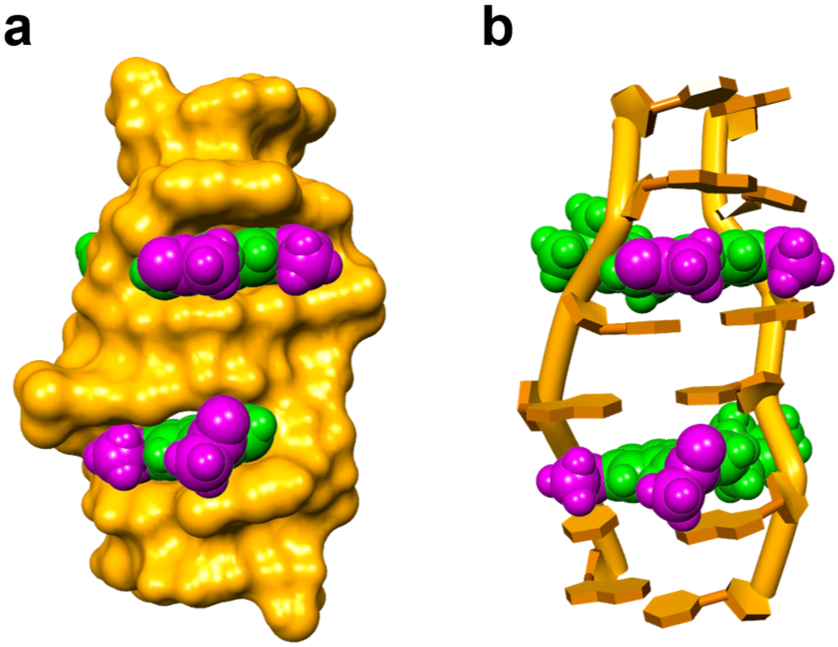

Figure S13. Molecular model show the acetyl groups (purple, CPK presentation) at the position of 3 and 7 of carbazole (green, CPK presentation) protruded into the major groove of Z-form duplex (orange) in solvent custom surface (a) or ribbon presentation (b), observed at major groove.

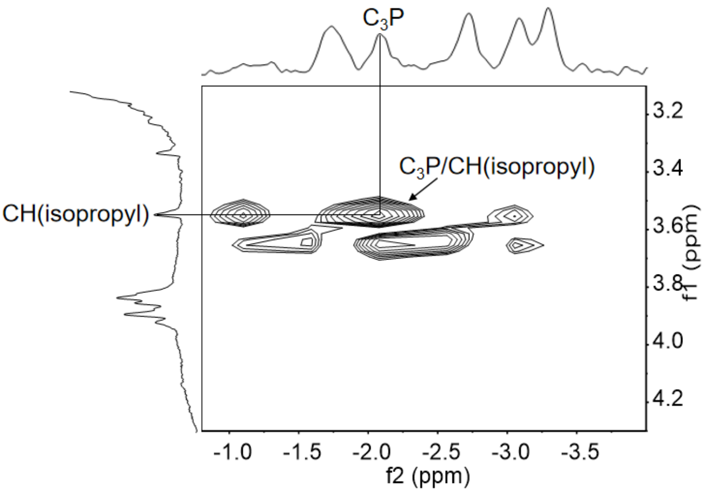

Figure S14. 2D heteronuclear ^{31}P-^{1}H NMR spectroscopy of Z-form RNA L-r(CGC^{F}GCG)$_2$ and CBL0137 complex at 20 °C in H_2O/D_2O (9:1), showing the cross peak between phosphorus of C_3 and CH(isopropyl) in CBL0137. Condition: 0.3 mM RNA in 10 mM Na-PO_4 and 2 M $NaClO_4$ at pH 7.0, with [RNA duplex]/[CBL0137] ratios in 1:2.

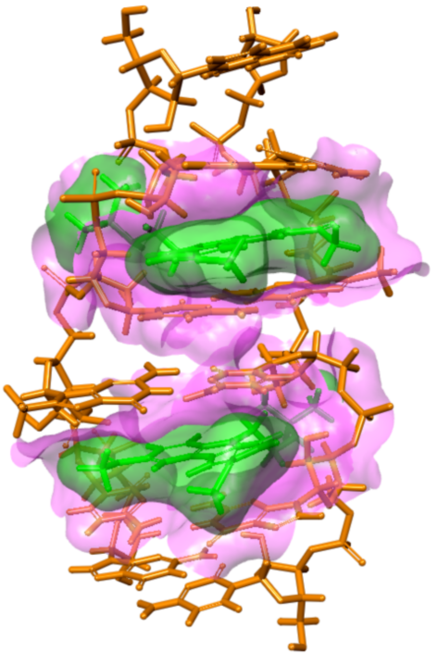

Figure S15. Molecular model of Z-form L-r(CGC^{F}GCG)$_2$/CBL0137 complex shows solvent hydrophobicity surface formation (purple) between RNA duplex (orange in stick) and two CBL0137 molecules (in green stick representation) surrounded in electronic cloud (green) and positioned in the cleft pocket.

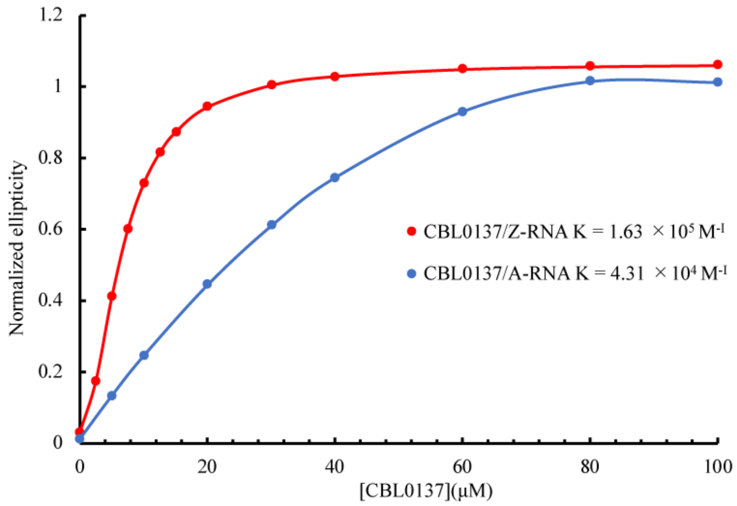

Figure S16. Titration data of CBL0137 and RNA derived by monitoring the wavelength at 280 nm of CD. The solid lines are the theoretical fit of the data. The binding constants of CBL0137 and right-handed Z-form RNA, CBL0137 and right-handed A-form RNA are indicated.

2',3',5'-tri-*O*-acetyl-L-guanosine (1)

L-Guanosine (500 mg, 1.76 mmol), trimethylamine (2.0 mL, 14.0 mmol) and 4-dimethylaminopyridine (25 mg, 0.2 mmol) were dissolved sequentially in 7 mL anhydrous acetonitrile under inert atmosphere, then acetic anhydride (0.4 mL, 5.6 mmol) was added dropwise, and the reaction mixture was stirred for 1.5 hours at 0 ℃ and another 1 hour at room temperature to complete the reaction. The reaction was quenched by additional methanol (0.56 mL, 14.43 mmol). By using a rotary evaporator to reduce the volume to 1/3, then diethyl ether was added dropwise to induce precipitation of a fine white solid. The crude product was filtered, washed with diethyl ether, and then stirred for 2 hours in acetone (6 mL) at 50 ℃. The filtrate produced 676.6 mg (94%) of a fine white powder. ^{1}H NMR (400 MHz, DMSO-d6) δ 10.73 (s, 1H), 7.94 (s, 1H), 6.55 (s, 2H), 5.99 (d, J = 8.0 Hz, 1H), 5.80 (t, J = 8.0 Hz, 1H), 5.50 (dd, J = 4.0 Hz, 1H), 4.41-4.25 (m, 3H), 2.12-2.05 (m, 9H); HRMS (ESI) for C22H35N6O8 $[M+H]^+$: Calcd. 409.1224; Found. 410.1337.

2’,3’,5’-tri-*O*-acetyl-8-trifluoromethyl-L-guanosine (2)

Dried 2',3',5'-tri-O-acetyl-L-guanosine (600 mg, 1.46 mmol) and zinc trifluoromethanesulfinate (1500 mg, 4.06 mmol) were dissolved in dimethyl sulfoxide (8 ml) and vigorous stirring till the clear solution was formed. Tert-butyl hydroperoxide (70% solution in water, 0.85 ml, 6.4 mmol) was added dropwise respectively in 10 aliquots (0.085 ml each) in a 20 min interval. The reaction mixture gradually turned yellow and continue reacted for 24 hours at room temperature under argon. The mixture was quenched by additional 100 mL water, and diluted in dichloromethane, extracted by 5% sodium bicarbonate and brine. The combine organic phases were dried over anhydrous sodium sulfate. The oily residue was purified by MPLC (medium pressure liquid chromatography) with the mixture of methanol in dichloromethane (5%, v/v) to give product 2 (315 mg, 50%). ^{1}H NMR (400 MHz, DMSO-d6) δ 12.20 (s, 1H), 6.29 (s, 1H),

5.95 (t, J = 6.4 Hz, 2H), 4.55-4.52 (m, 1H), 4.46-4.38 (m, 2H), 2.15-2.04 (m, 9H); 19F NMR (372 MHz, DMSO-d6) δ -61.1843 (s, 3F); HRMS (ESI) for C17H15O8N5F3Na $[M+Na]^+$: Calcd. 477.1107; Found. 500.0981.

8-trifluoromethyl-L-guanosine (3)

2',3',5'-tri-O-acetyl-8-trifluoromethyl-L-guanosine (400 mg, 0.838 mmol) was treated with methylamine (33% in ethanol, 4.44 mL, 42.56 mmol), and the mixture was stirred for 4 hours at room temperature under argon atmosphere. The reaction mixture then was concentrated, and the residue was purified by MPLC with the mixture of methanol in dichloromethane (10%, v/v). The product 3 was giving as yellowish solid (274.7 mg, 94%). ^{1}H NMR (400 MHz, DMSO-d6) δ 11.05 (s, 1H), 6.72 (s, 2H), 5.64 (d, J = 6.0 Hz, 1H), 5.50 (d, J = 4.4 Hz, 1H), 5.14-4.92 (m, 3H), 4.17 (dd, J = 4.8 Hz, 1H), 3.91 (dd, J = 3.2 Hz, 1H), 3.71-3.52 (m, 2H); 19F NMR (372 MHz, DMSO-d6) δ -59.8351 (s, 3F); HRMS (ESI) for C11H11O5N5F3 $[M-H]^-$: Calcd. 350.0688; Found. 350.0695.

N^2-dimethylformamidyl-8-trifluoromethyl-L-guanosine (4)

8-trifluoromethyl-L-guanosine (500 mg, 1.43 mmol) and N,N-dimethylformamide dimethyl acetal (1.36 mL, 10.1 mmol) were added sequentially to the solution of anhydrous dimethylformamide (10 mL). The mixture was reacted for 1 hour at room temperature under argon and the solvent was evaporated to get residue. The crude product was purified by MPLC with mixture of methanol in dichloromethane (20%, v/v), the product 4 was giving as white foam (475 mg, 82%). ^{1}H NMR (400 MHz, DMSO-d6) δ 11.76 (s, 1H), 8.54 (s, 1H), 5.68 (d, J = 6.0 Hz, 1H), 5.42 (d, J = 6.4 Hz, 1H), 5.24 (d, J = 4.8 Hz, 1H), 5.05 (J = 6.0 Hz, 1H), 4.89 (J = 4.6 Hz, 1H), 4.27 (dd, J = 5.2 Hz, 1H), 3.94 (dd, J = 3.6 Hz, 1H), 3.72-3.56 (m, 2H), 3.18 (s, 3H), 3.08 (s, 3H); HRMS (ESI) for C14H16O5N6F3 $[M-H]^-$: Calcd. 405.1231; Found. 405.1225.

N2-dimethylformamidyl-8-trifluoromethyl-2'-O-(tert butyldimethylsilyl) 3', 5'-O-(di-tert butylsilylene)-L-guanosine (5)

N2-dimethylformamidyl-8-trifluoromethyl-L-guanosine (200 mg, 0.48 mmol) was dissolved in anhydrous dimethylformamide (2 mL), and di-tert-butylsilyl bis (trifuoromethanesulfonate) (230 mg, 0.53 mmol) was added dropwise, and cooled down in an ice-bath under argon. The mixture reacted for 15 minutes at 0 °C. Imidazole (163.5 mg, 2.4 mmol) also was added, and the mixture continue reacted for 15 minutes at 0 °C and another 15 minutes at room temperature. tert-butyldimethylsilyl chloride (345.6 mg, 2.3 mmol) was added, the reaction mixture reacted for 4 hours at 60 °C. The reaction solvent was evaporated in vacuo and the residue was purified by MPLC with mixture of chloroform in ethyl acetate (25%, v/v). The product was giving as white foam (260 mg, 82%). ^{1}H NMR (400 MHz, DMSO-d6) δ 9.19 (s, 1H), 8.38 (s, 1H), 8.01 (s, 1H), 5.82 (d, J = 1.2 Hz, 1H), 5.09 (dd, J = 2.0 Hz, 1H), 4.52 (dd, J = 6.0 Hz, 1H), 4.42 (dd, J = 4.8 Hz, 1H), 4.06 (dt, J = 5.2 Hz, 1H), 3.93 (t, J = 6.8 Hz, 1H), 3.18-3.14 (m, 7H), 2.95-2.87 (m, 6H), 1.68 (s, 6H), 1.06-0.86 (m, 45H), 0.08-0.04 (m, 12H); HRMS (ESI) for C28H48O5N6F3Si2Na $[M+Na]^+$: Calcd. 661.3099; Found. 683.2984.

N2-dimethylformamidyl-8-trifluoromethyl-5'-*O*-(4,4'dimethoxytrityl)-2'-*O*-tert-

butyldimethylsilyl-L-guanosine (6)

Compound 5 (500 mg, 0.75 mmol) was dissolved in 2 mL dichloromethane, cooled down in an ice-bath under argon. 83 µL hydrofluoric acid-pyridine solution (70% hydrofluoric acid, 30% pyridine) diluted in 0.52 mL pyridine was then added, stirred at 0 ℃ for 2 hours. The mixture was extracted by dichloromethane and combined organic layer evaporated, giving crude product 390 mg. No further purification, the crude product (390 mg) and 4,4'-dimethoxytrityl chloride (362.4 mg, 1.07 mmol) were dissolved in 3 mL anhydrous pyridine and the mixture stirred for 4 hours at room temperature. The solvent was concentrated in vacuo and the residue was purified by MPLC with mixture of dichloromethane in ethyl acetate (25%, v/v). The product was giving as white foam (370 mg, 60%). ^{1}H NMR (400 MHz, DMSO-d6) δ 9.05 (s, 1H), 8.61 (dt, J = 12 Hz, 1H), 8.20 (s, 1H), 7.41-7.14 (m, 8H), 6.77-6.73 (m, 4H), 5.81 (d, J = 4.0 Hz, 1H), 5.14 (dd, J = 4.0 Hz, 1H), 4.54 (dd, J = 6.4 Hz, 1H), 4.02 (dd, J = 12.4 Hz, 1H), 3.76 (s, 6H), 3.44-3.40 (m, 2H), 2.99 (s, 3H), 2.63 (s, 3H), 2.04 (s, 1H), 0.86 (s, 10H), 0.118 (s, 3H), 0.008 (s, 3H); HRMS (ESI) for C41H50O7N6F3Si $[M+H]^+$: Calcd. 823.3384; Found. 823.3445.

3'-*O*-phosphoramidite-N^2-dimethylformamidyl-8-trifluoromethyl-5'-*O*-(4,4'-dimethoxytrityl)-2'-*O*-tert- butyldimethylsilylguanosine (7)

Compound 6 (800 mg, 0.97 mmol) co-evaporated with 5 mL anhydrous acetonitrile by three times and dissolved in 7 mL anhydrous dichloromethane. Diisopropylethylamine (0.62 mL, 3.6 mmol) and 1-methylimidazole (0.07 mL, 0.9 mmol) were added under argon atmosphere. After 5 minutes, 2-cyanoethyl-N,N-diisopropylamidochlorophosphoramidite (0.6 mL, 2.94 mmol) was then added dropwise over 5 min, the mixture was stirred for 1.5 hours at room temperature. The reaction mixture was extracted by dichloromethane and organic layer concentrated and purified by MPLC with mixture of ethyl acetate in dichloromethane (25%, v/v). The product was giving as white foam (730 mg, 67%). ^{1}H NMR (400 MHz, DMSO-d6) δ 8.76 (s, 1H), 8.03 (s, 1H), 7.92 (s, 1H), 7.48-7.17 (m, 12H), 6.78-6.72 (m, 5H), 5.91-5.88 (m, 1H), 5.30 (m, 1H), 4.58-4.31 (m, 2H), 4.11 (s, 1H), 3.76-3.47 (m, 16H), 2.98-2.56 (m, 5H), 2.31 (s, 1H), 2.10-2.02 (m, 2H), 1.29-0.73 (m, 30H), 0.08-0.00 (m, 11H); ^{31}P NMR (161 MHz, DMSO-d6) δ 150.86, 148.17.

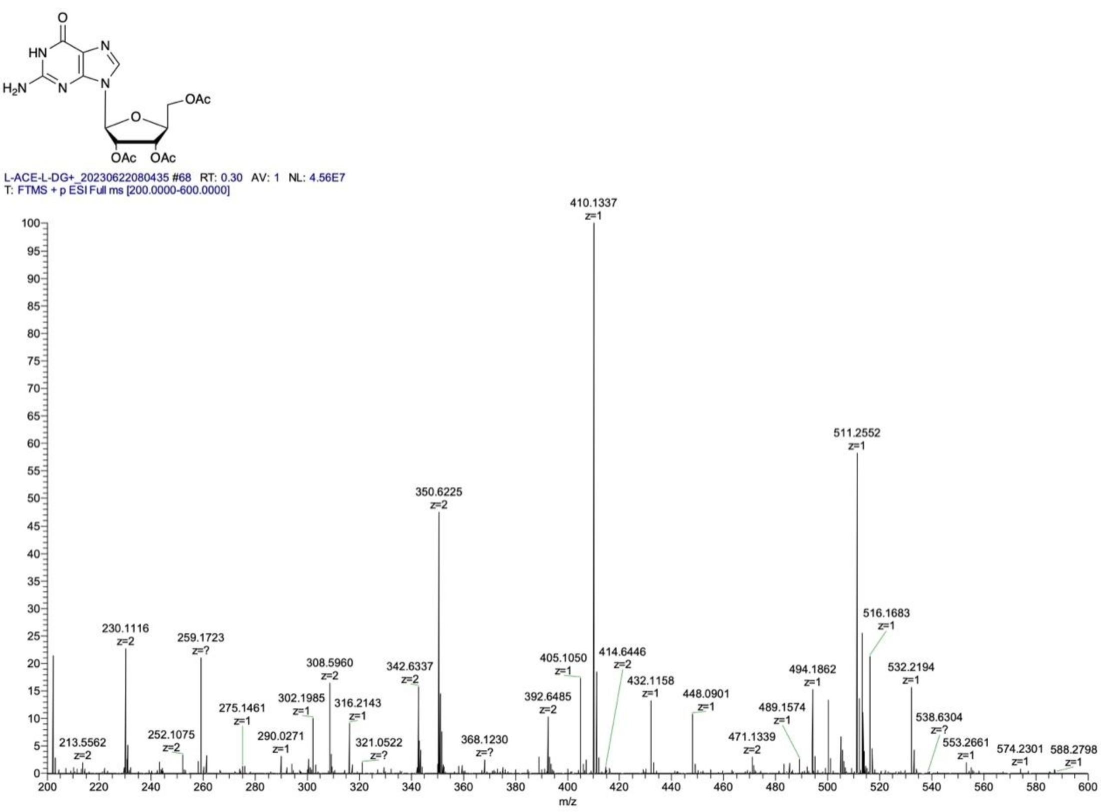

Data S1. ESI-MS spectrum of compound **1**.

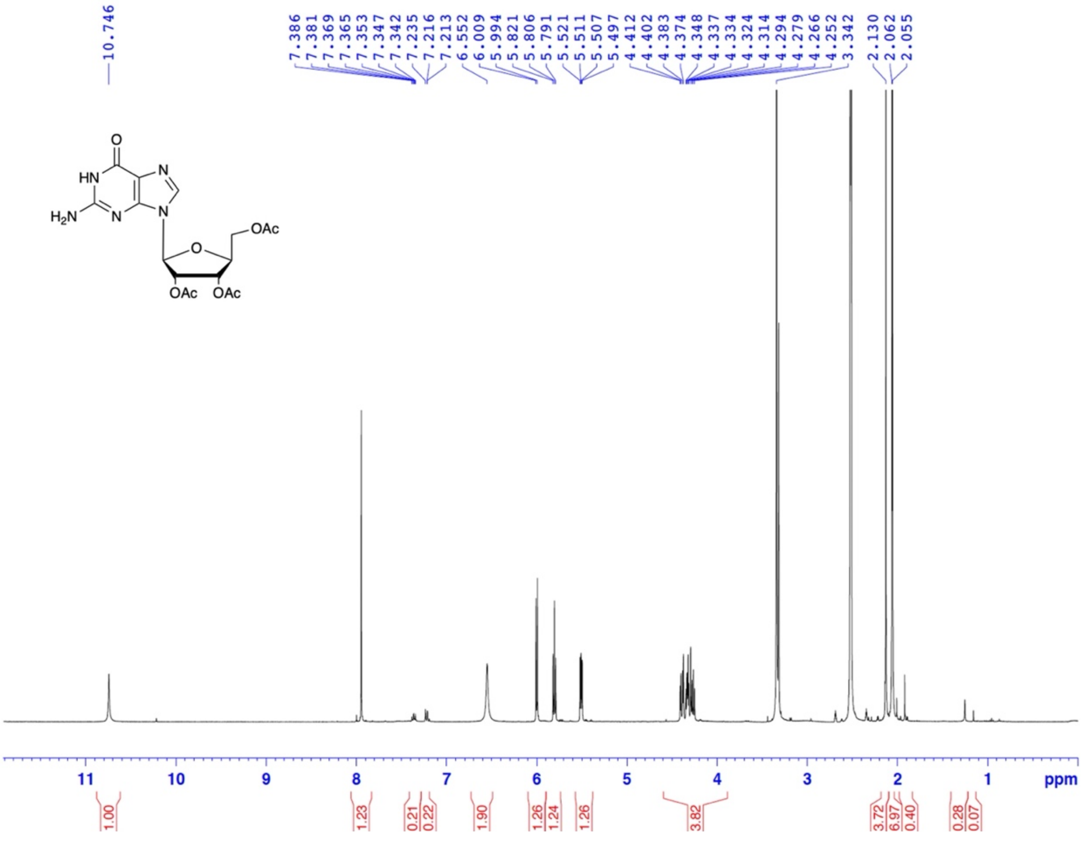

Data S2. ^{1}H NMR spectrum of compound **1**.

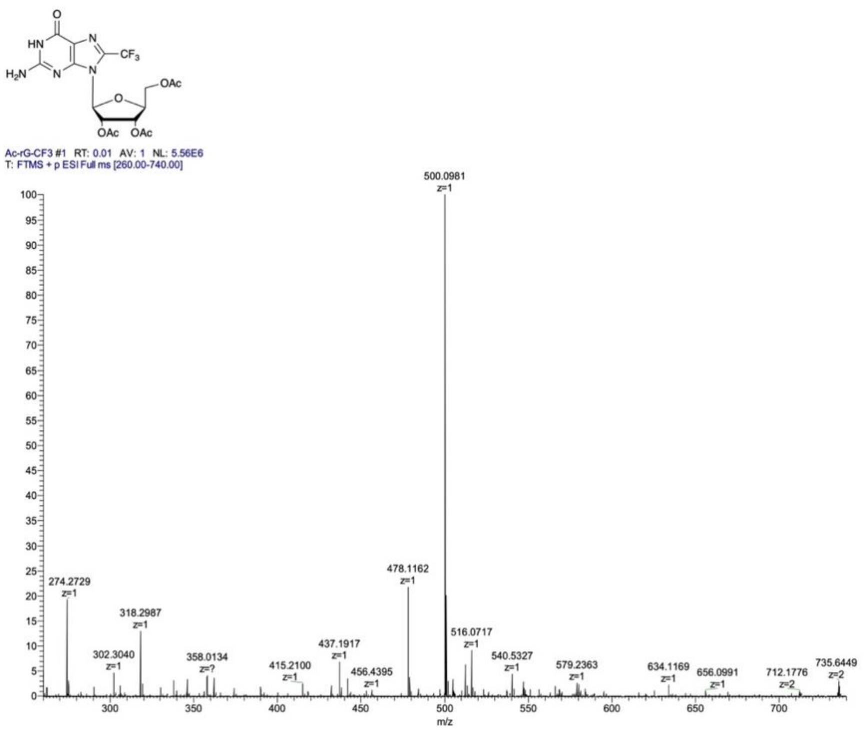

Data S3. ESI-MS spectrum of compound **2**.

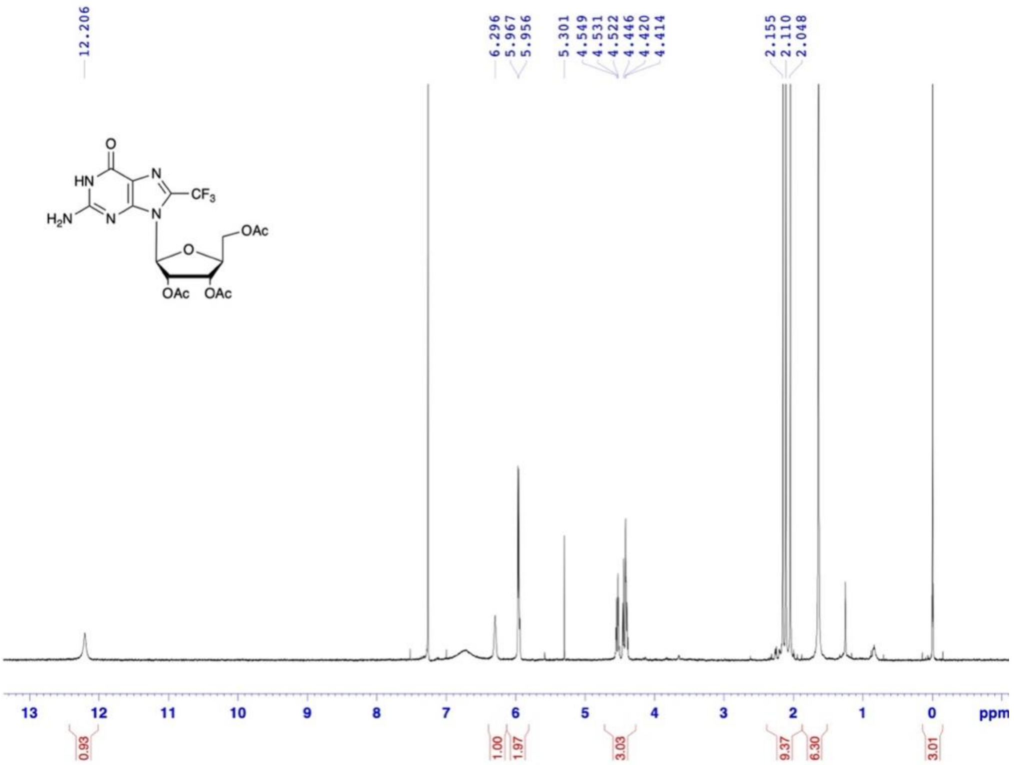

Data S4. ^{1}H NMR spectrum of compound **2**.

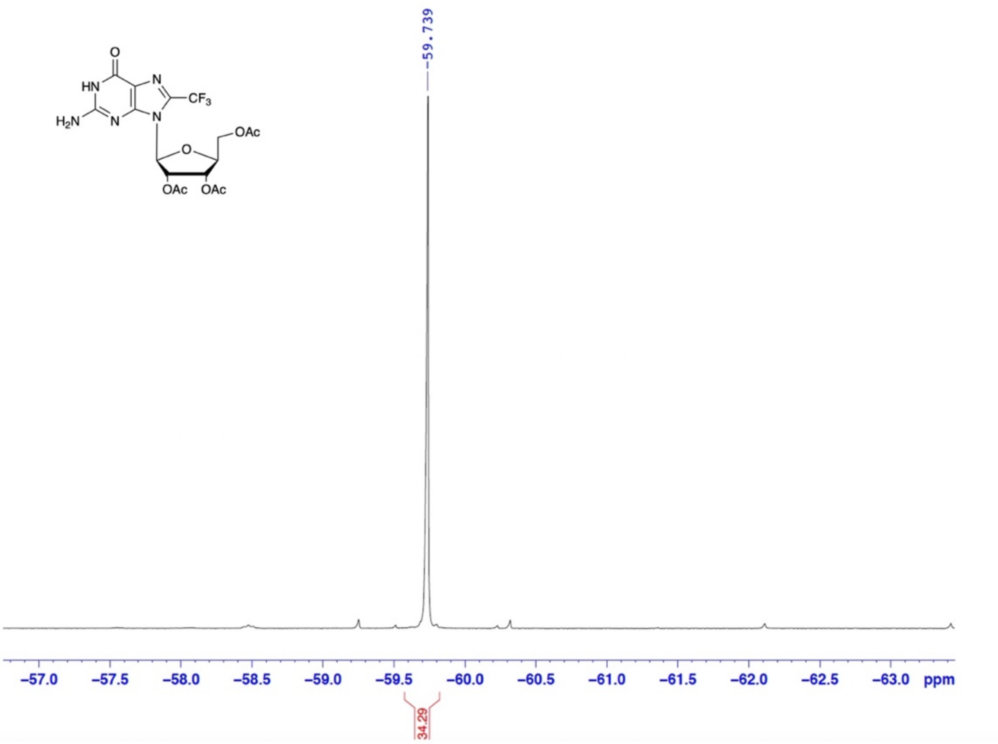

Data S5. ^{19}F NMR spectrum of compound **2**.

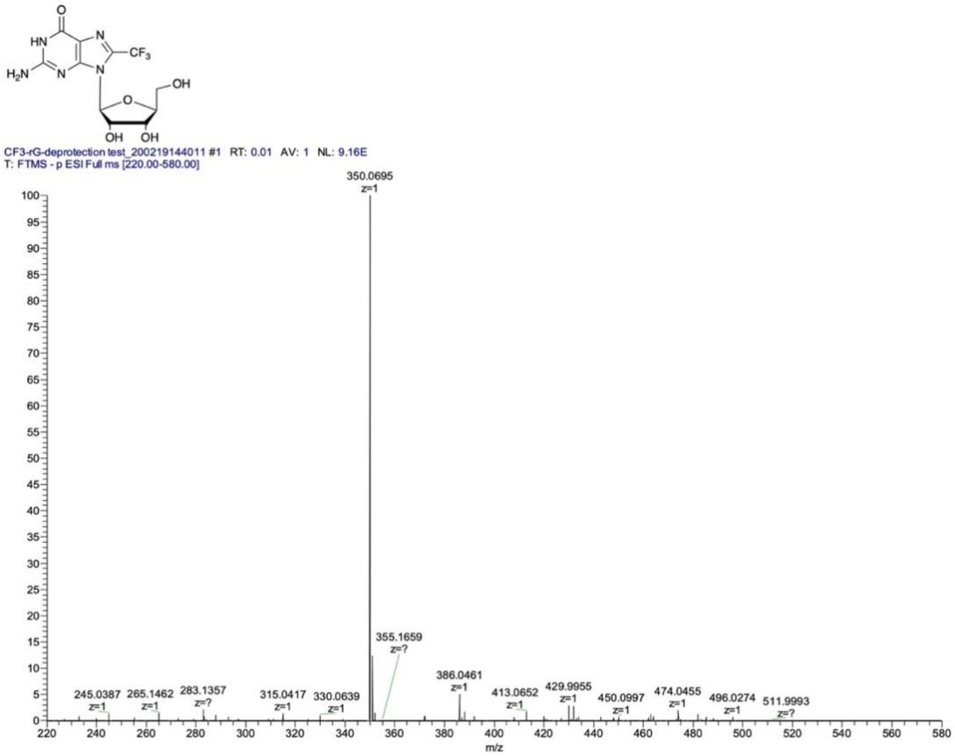

Data S6. ESI-MS spectrum of compound **3**.

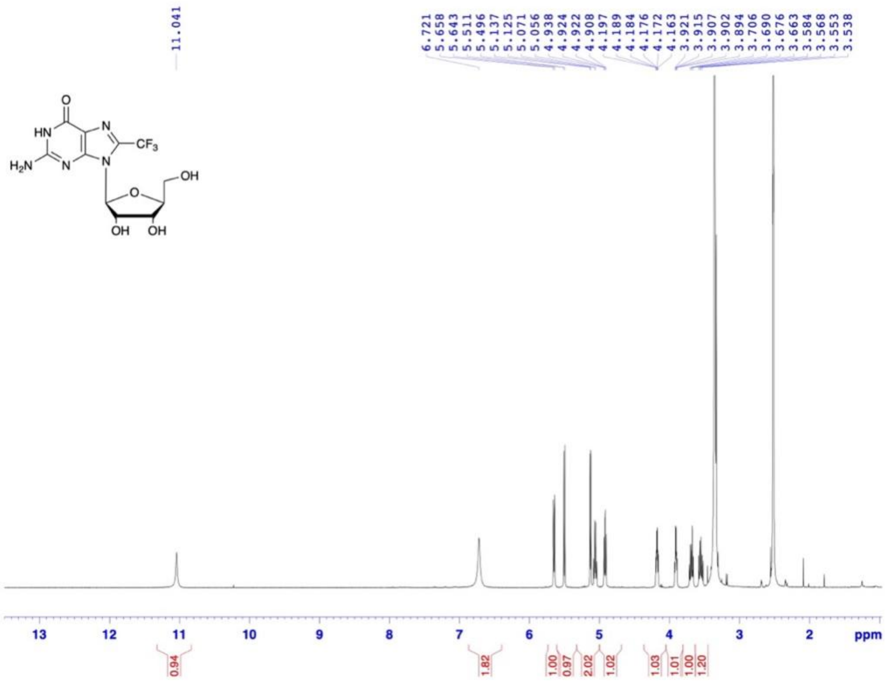

Data S7. ^{1}H NMR spectrum of compound **3**.

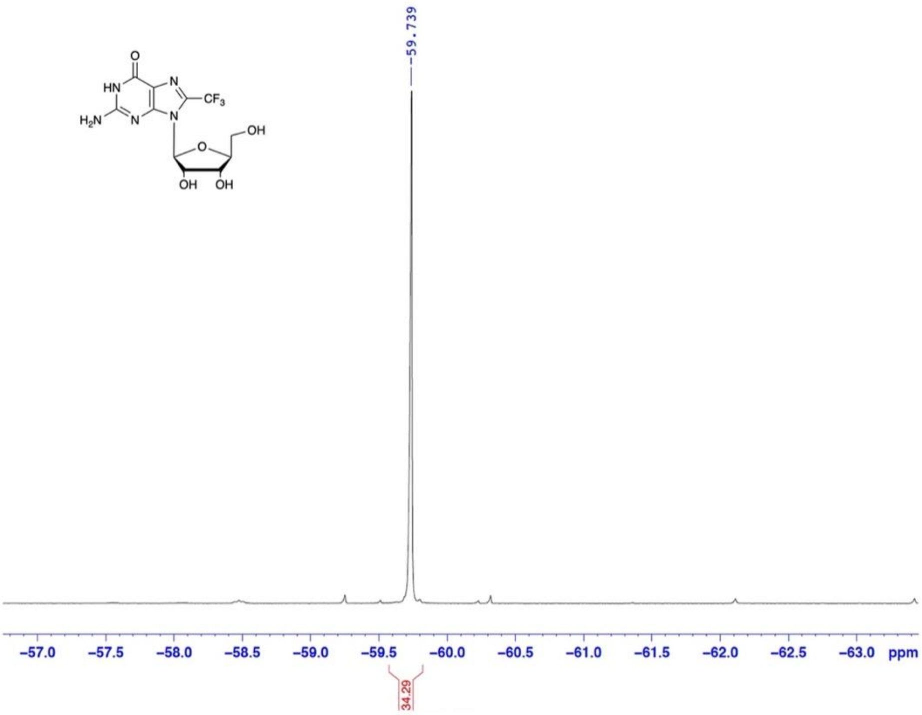

Data S8. ^{19}F NMR spectrum of compound **3**.

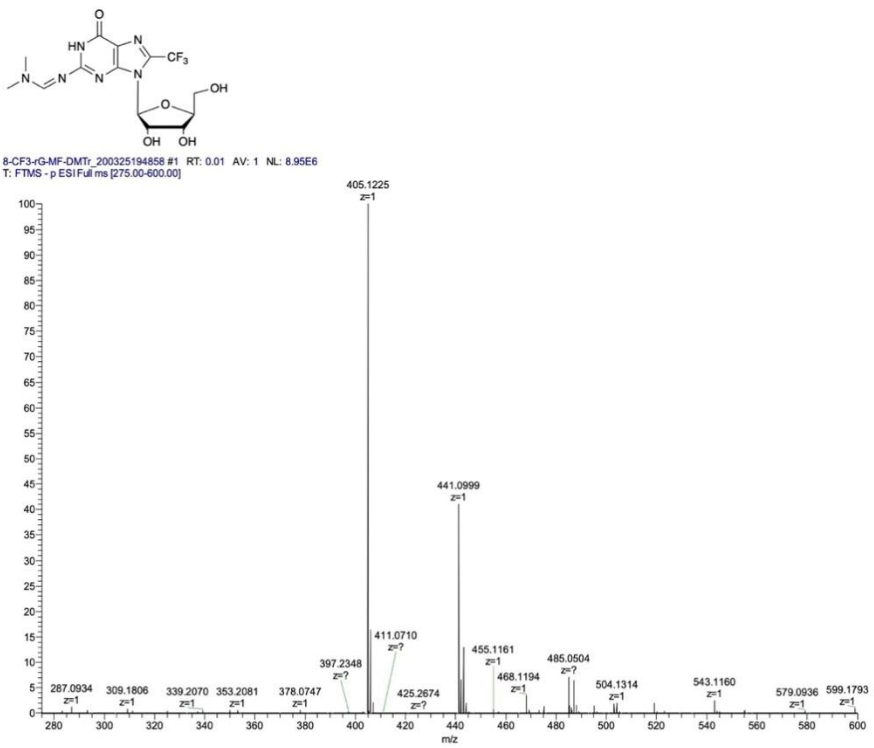

Data S9. ESI-MS spectrum of compound **4**.

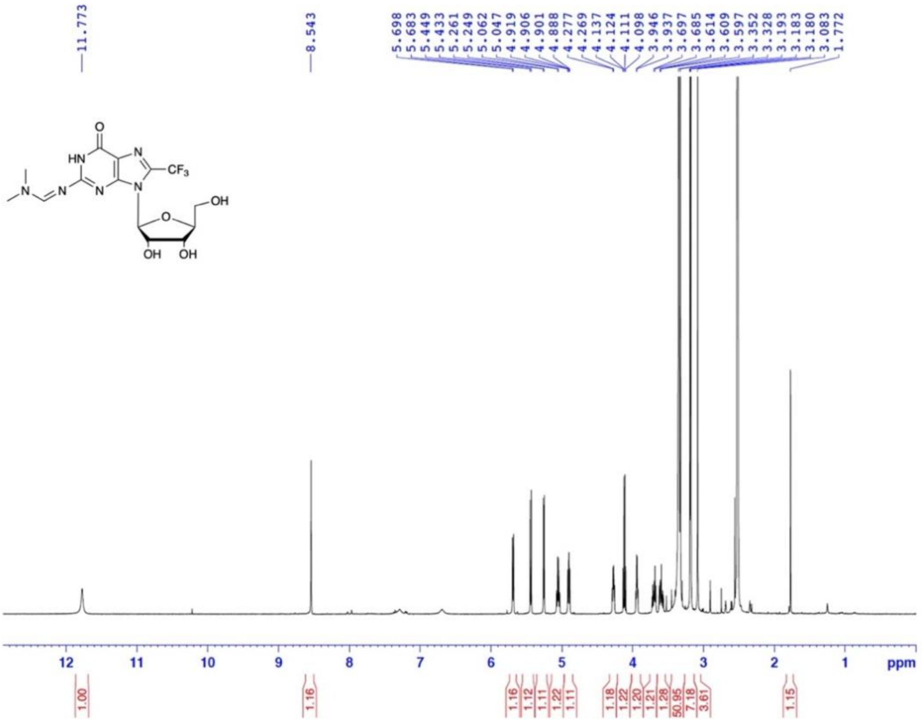

Data S10. ^{1}H NMR spectrum of compound **4**.

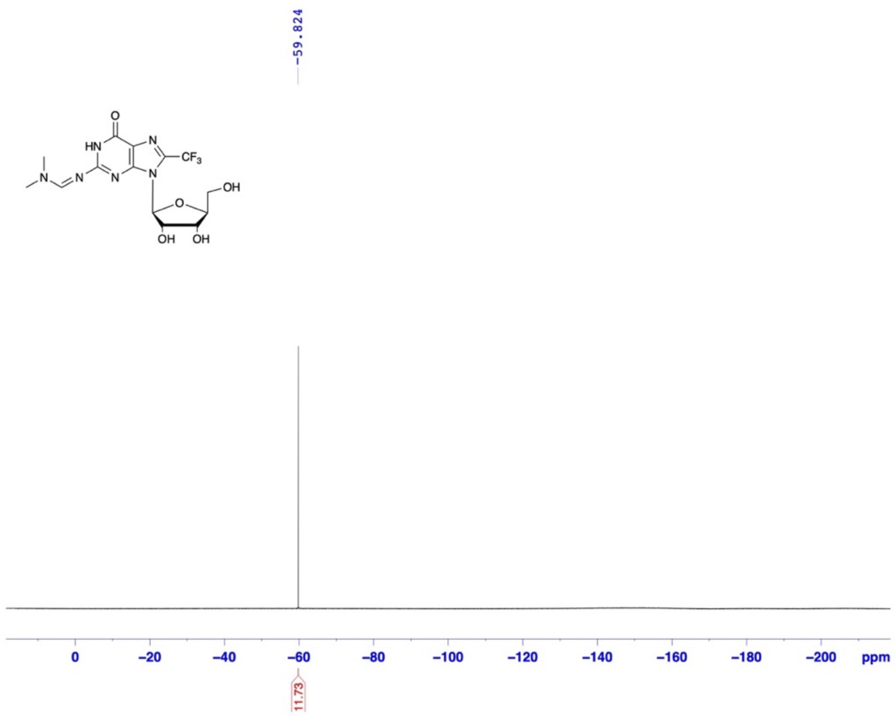

Data S11. ^{19}F NMR spectrum of compound **4**.

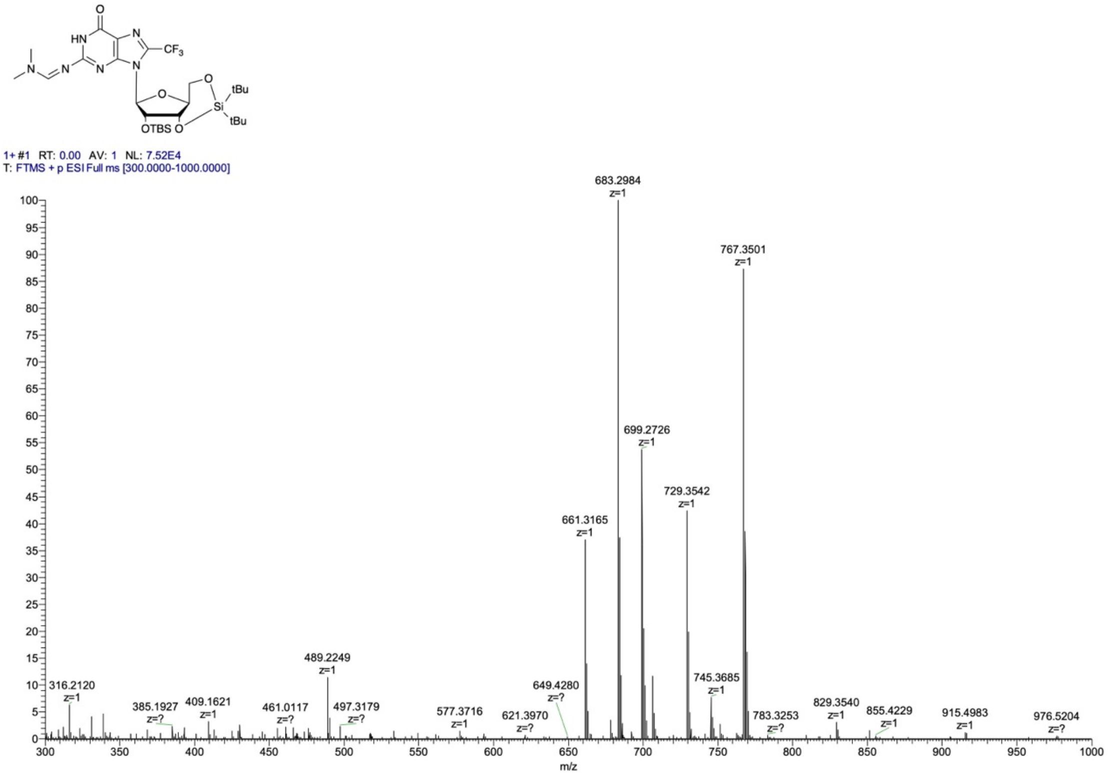

Data S12. ESI-MS spectrum of compound **5**.

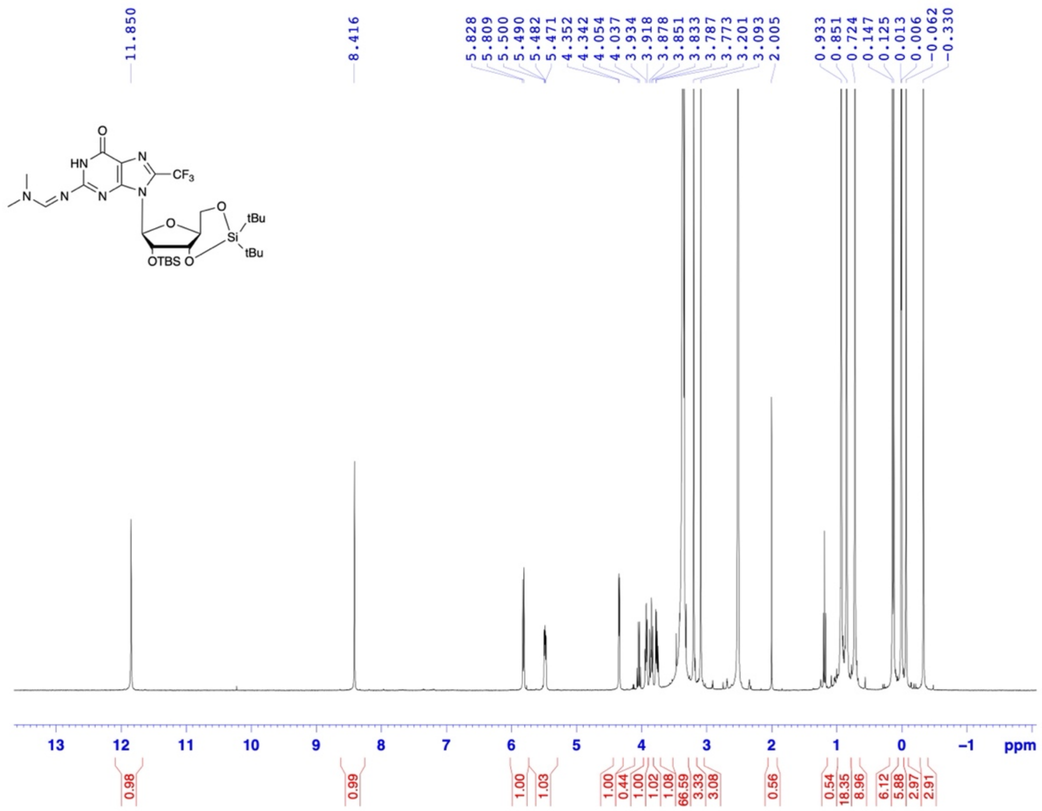

Data S13. ^{1}H NMR spectrum of compound **5**.

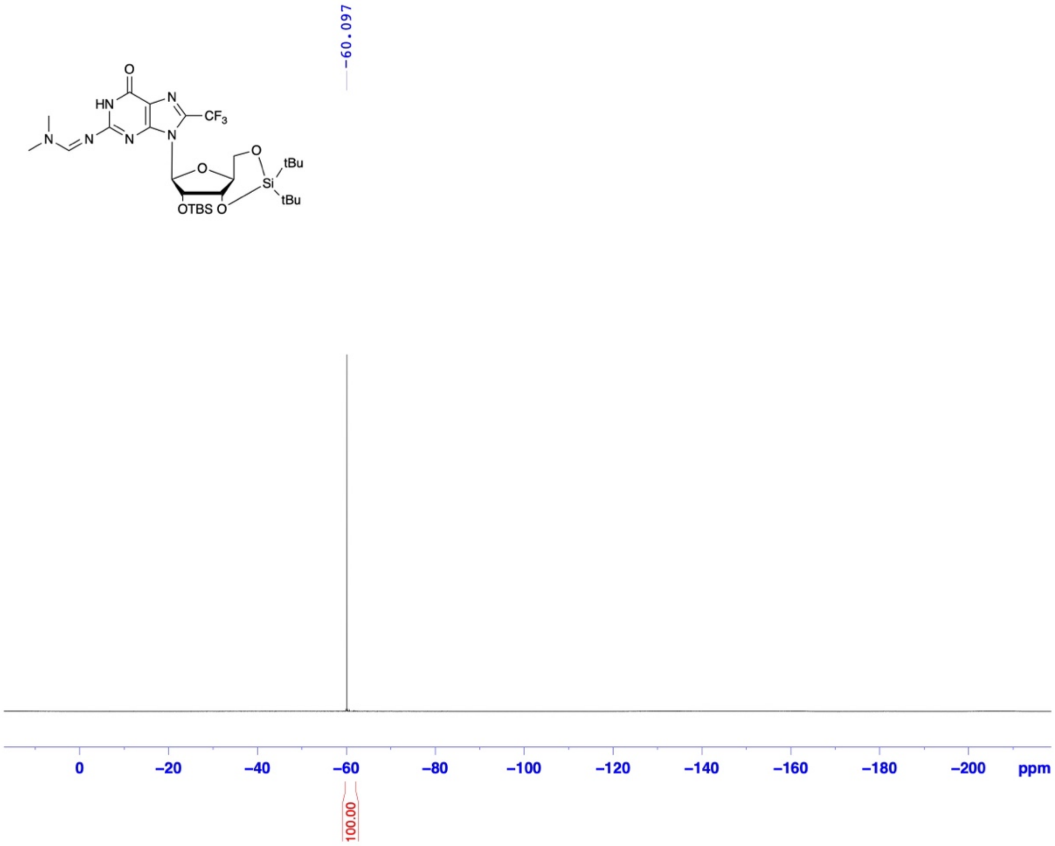

Data S14. ^{19}F NMR spectrum of compound **5**.

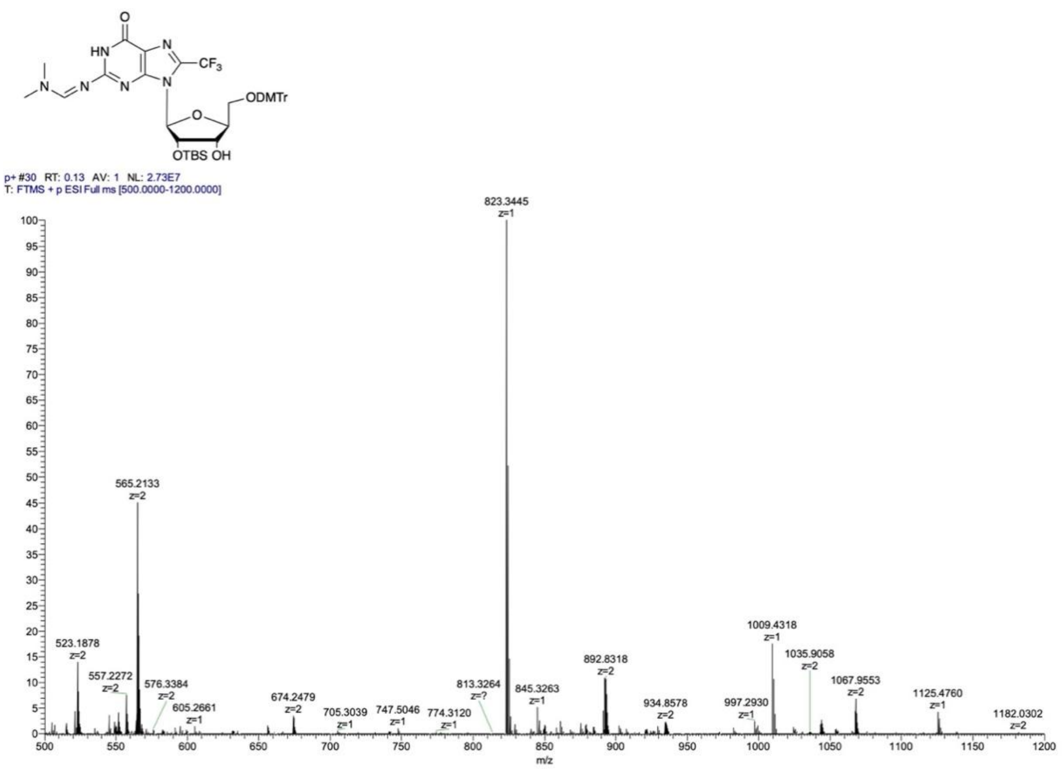

Data S15. ESI-MS spectrum of compound **6**.

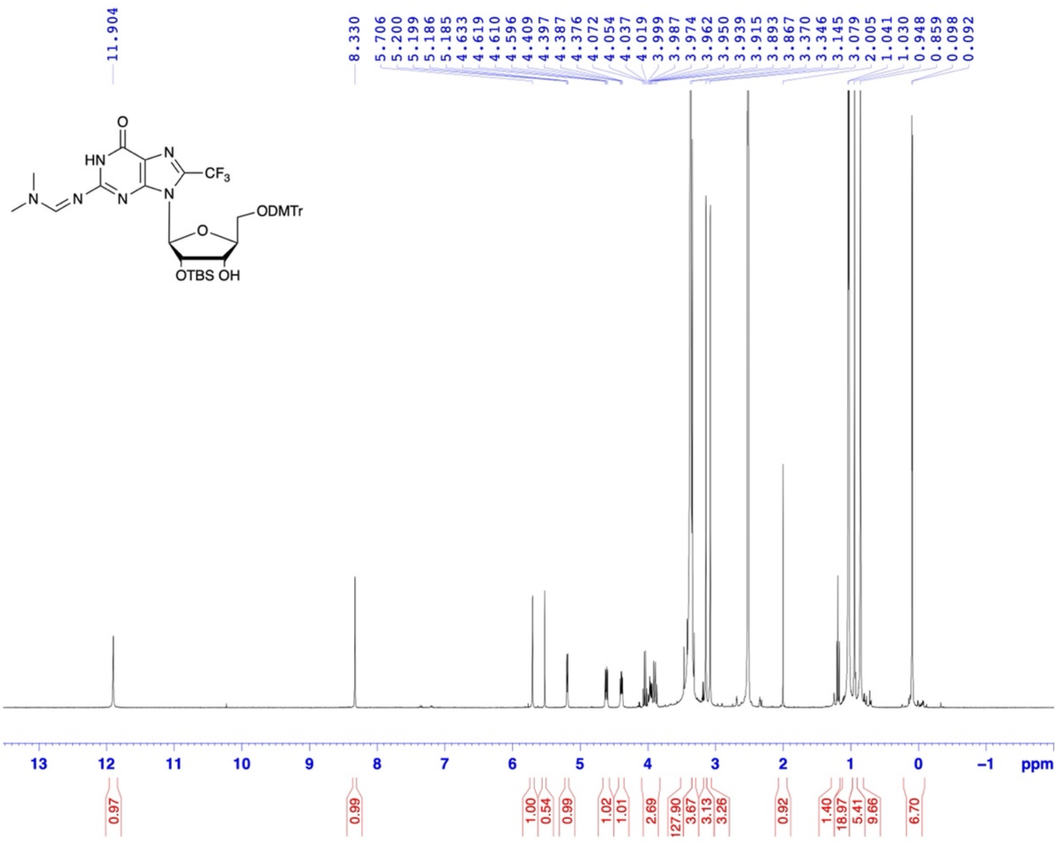

Data S16. ^{1}H NMR spectrum of compound **6**.

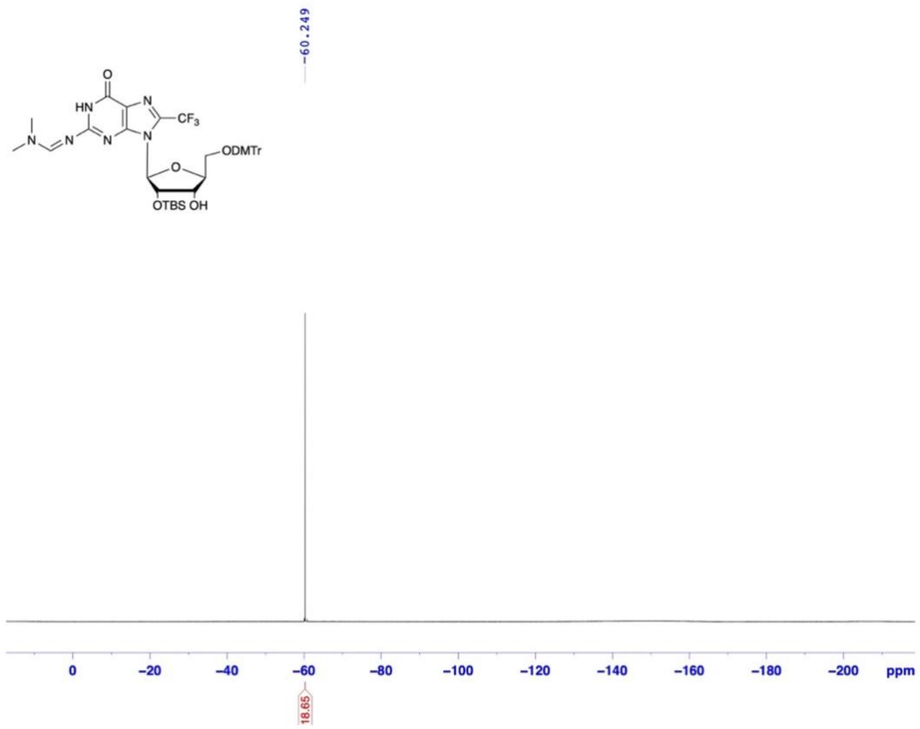

Data S17. ^{19}F NMR spectrum of compound **6**.

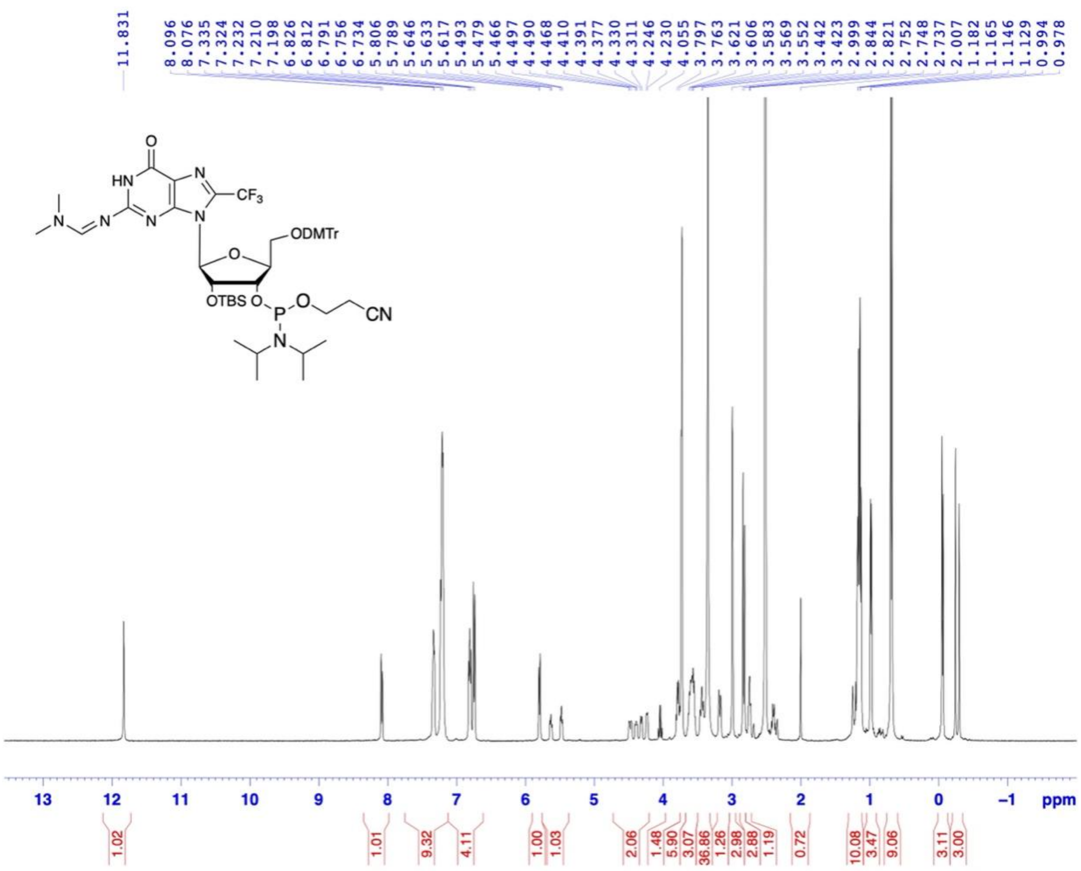

Data S18. ^{1}H NMR spectrum of compound **7**.

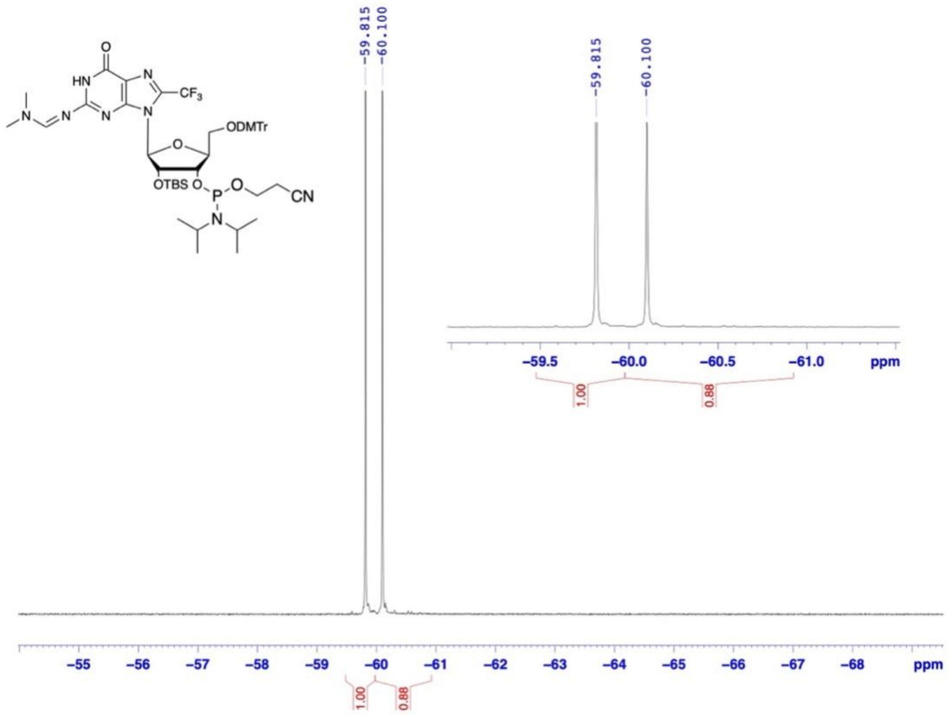

Data S19. ^{19}F NMR spectrum of compound **7**.

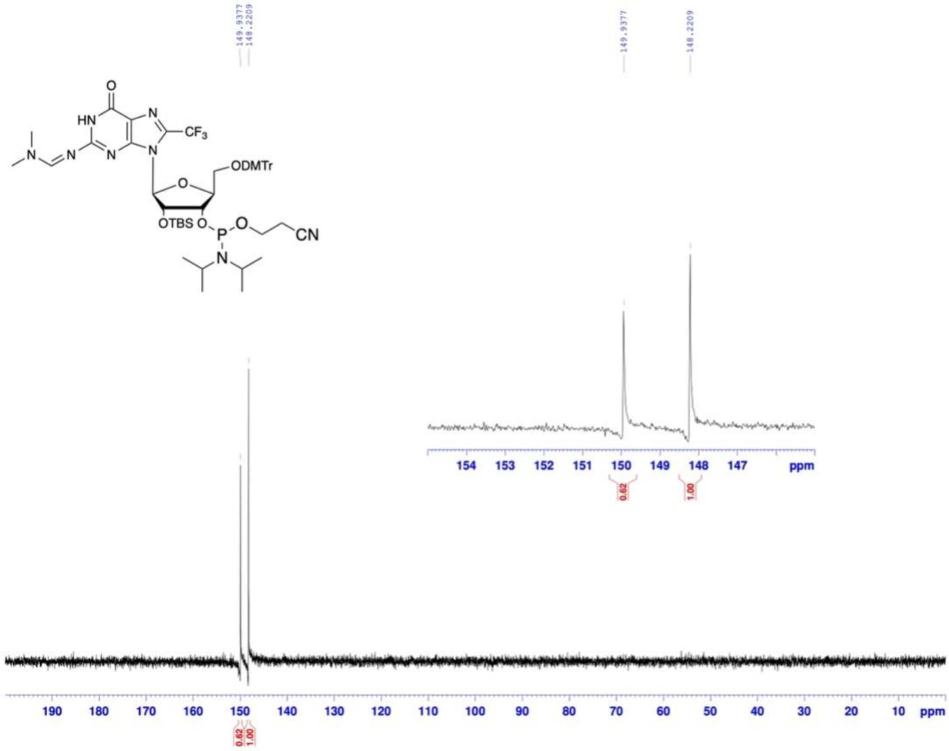

Data S20. ^{31}P NMR spectrum of compound **7**.

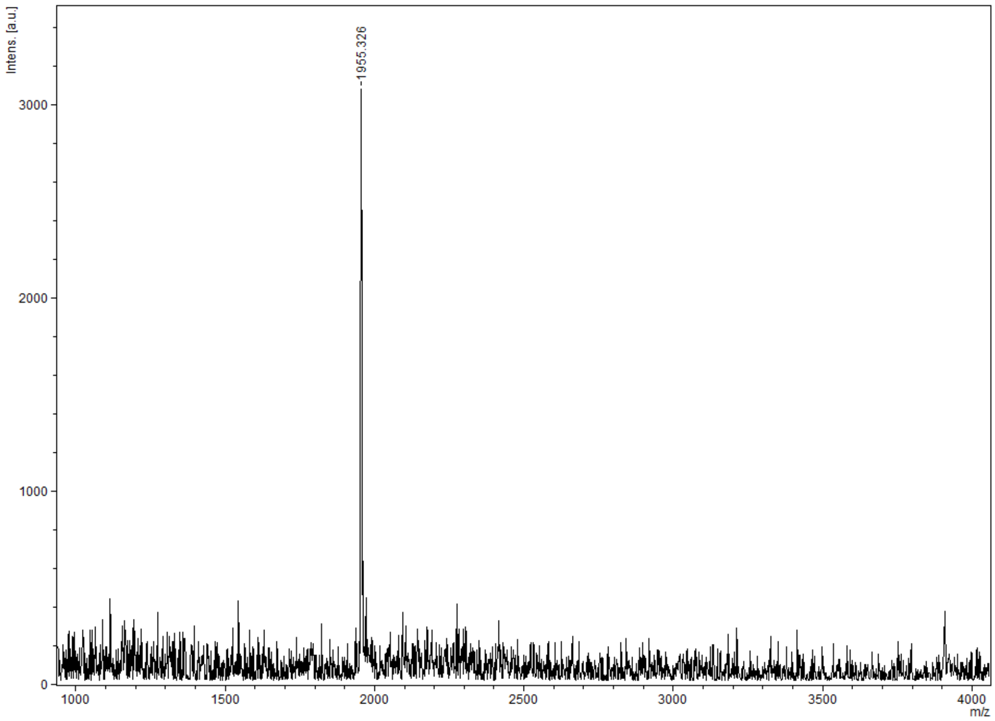

Data S21. MALDI TOF MS of L-CGC^{F}GCG.

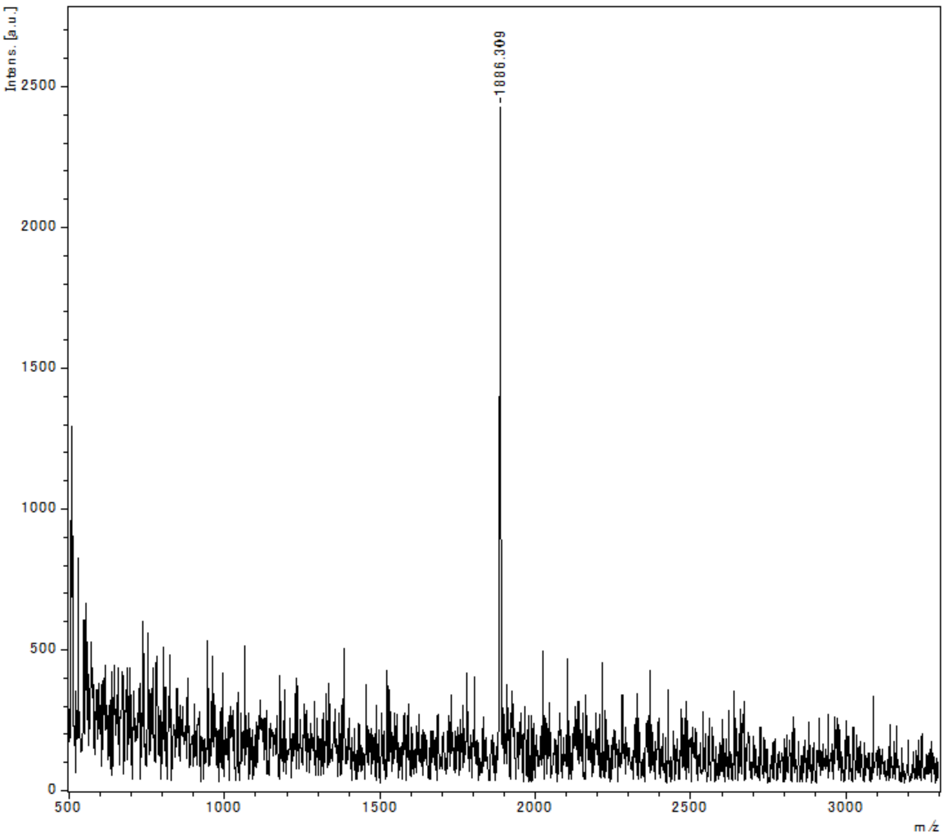

Data S22. MALDI TOF MS of L-CGCGCG.

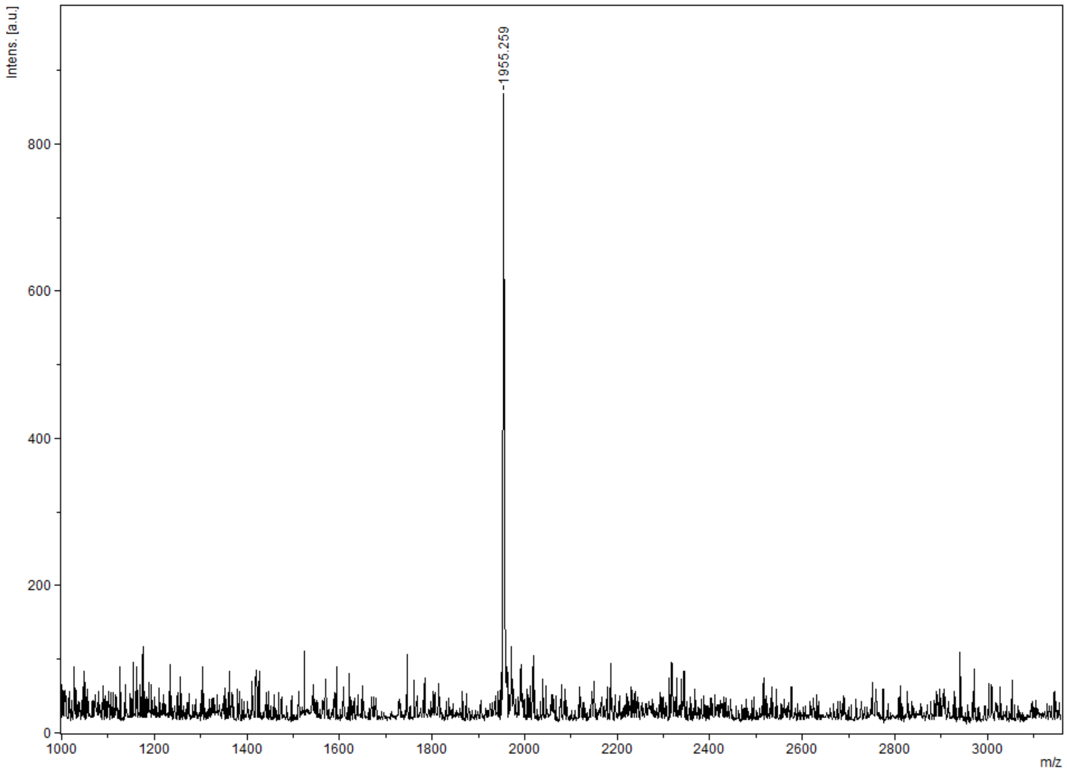

Data S23. MALDI TOF MS of D-CGC^{F}GCG.

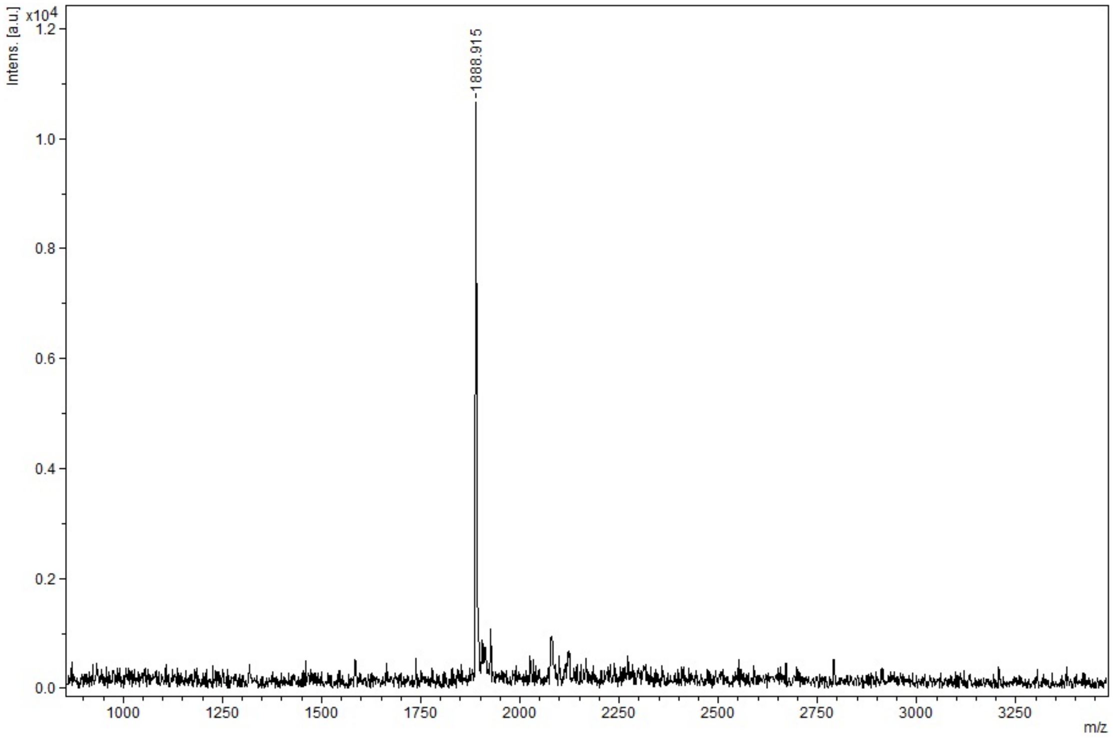

Data S24. MALDI TOF MS of D-CGCGCG.
